# Phospholipid Profiles Are Selectively Altered in the Putamen and White Frontal Cortex of Huntington’s Disease

**DOI:** 10.3390/nu14102086

**Published:** 2022-05-16

**Authors:** Gabrielle R. Phillips, Sarah E. Hancock, Andrew M. Jenner, Catriona McLean, Kelly A. Newell, Todd W. Mitchell

**Affiliations:** 1 Illawarra Health and Medical Research Institute, University of Wollongong, Wollongong 2522, Australia; gphillip@uow.edu.au (G.R.P.); knewell@uow.edu.au (K.A.N.); 2 School of Medical, Indigenous and Health Sciences, Faculty of Science, Medicine and Health, University of Wollongong, Wollongong 2522, Australia; 3 Molecular Horizons, University of Wollongong, Wollongong 2522, Australia; 4 School of Medical Sciences, University of New South Wales, Sydney 2052, Australia; s.hancock@victochang.edu.au; 5 Bioanalytical Mass Spectrometry Facility, Mark Wainwright Analytical Centre, University of New South Wales, Sydney 2052, Australia; andrew.jenner@unsw.edu.au; 6 Department of Anatomical Pathology, Alfred Health and Florey Neuroscience, Parkville 3052, Australia; catriona.mclean@monash.edu

**Keywords:** Huntington’s disease, phospholipids, cortex, striatum, white matter, lipids

## Abstract

Huntington’s disease (HD) is a genetic, neurodegenerative illness that onsets in late adulthood as a series of progressive and terminal cognitive, motor, and psychiatric deficits. The disease is caused by a polyQ mutation in the Huntingtin gene (*HTT*), producing a polyglutamine expansion in the Huntingtin protein (HTT). HTT interacts with phospholipids in vitro; however, its interactions are changed when the protein is mutated in HD. Emerging evidence suggests that the susceptibility of brain regions to pathological stimuli is influenced by lipid composition. This study aimed to identify *where* and *how* phospholipids are changed in human HD brain tissue. Phospholipids were extracted using a modified MTBE method from the post-mortem brain of 13 advanced-stage HD patients and 13 age- and sex-matched controls. Targeted precursor ion scanning mass spectrometry was used to detect phospholipid species. In the white cortex of HD patients, there was a significantly lower abundance of phosphatidylcholine (PC) and phosphatidylserine (PS), but no difference in phosphatidylethanolamine (PE). In HD putamen, ester-linked 22:6 was lower in all phospholipid classes promoting a decrease in the relative abundance of ester polyunsaturated fatty acids in PE. No differences in phospholipid composition were identified in the caudate, grey cortex or cerebellum. Ether-linked PE fatty acids appear protected in the HD brain, as no changes were identified. The nature of phospholipid alterations in the HD brain is dependent on the lipid (subclass, species, and bond type) and the location.

## 1. Introduction

Huntington’s disease (HD) is an autosomal, dominant, neurodegenerative illness resulting from a CAG repeat mutation on exon-1 of the Huntingtin gene (*HTT*). This mutation causes a polyglutamine expansion at the N-terminus of the huntingtin protein [1] (HTT), referred to as mutant huntingtin (mHTT). Although ubiquitously expressed in the human body, the presence of mHTT coincides with the targeted degeneration of several brain regions, notably the striatum and cerebral cortex. This degeneration corresponds with the progressive and terminal cognitive, psychiatric, and motor symptoms which onset in late adulthood in HD [2]. Because there is limited knowledge concerning how the mHTT causes the selective degeneration of these brain regions, no effective treatment options are available. HTT is hypothesized to be critical to neurodevelopment [3,4], and appears to play important roles in multiple cellular processes, including synaptic development [5], transcriptional regulation [6], autophagy [6,7], and neuronal survival [5]. However, despite brain atrophy being the dominant feature of HD, multiple peripheral pathologies have been identified in human embryonic stem cells [8], skeletal muscle [9], and cardiomyocytes (via central nervous system stimulation) [10,11], likely linked by defects in energy metabolism. 

Whether the neurodegeneration in HD results from a loss of function of HTT or a gain of function of mHTT is not well understood [3]. The length of the CAG repeat in the *HTT* gene is associated with disease onset [12,13]. Longer CAG repeat lengths are associated with an earlier onset; those over 60 determine juvenile onset [2,12]. Similarly, the polyglutamine length of the HTT protein is associated with neural lipid interactions [14,15,16,17]. HTT naturally associates and interacts selectively with phospholipids in neural cell cultures. These preferences and interactions are changed when HTT is mutated in HD. 

Phospholipids are a lipid class comprising two fatty acids attached to a phosphate head group and glycerol backbone. The phosphate head group distinguishes the phospholipids into one of six subclasses: phosphatidic acid (PA), phosphatidylcholine (PC), phosphatidylethanolamine (PE), phosphatidylglycerol (PG), phosphatidylinositol (PI) or phosphatidylserine (PS). Phospholipids are predominately cell membrane components, with PC, PE and PS contributing the more significant portion of the reserve in the human brain. Whilst the fatty acyl chains at the *sn*-2 position of PC and PE are attached by an ester bond, the fatty acyl chain at the *sn*-1 can be attached by an ester, vinyl ether or alkyl ether bond [18]. Phospholipids that contain vinyl ether bonds are plasmalogens [19]. The ether linkage allows the *sn*-1 fatty acyl chain to be arranged perpendicular to the molecule, unlike ester linkages which bend the chain. Because of this, plasmalogens form tighter packing in the cell membrane, decreasing its fluidity [20]. The ability of phospholipids to selectively release the fatty acyl chains at the *sn*-1 and *sn*-2 positions of the glycerol backbone allows them to contribute to the formation of second messengers, cell signalling molecules and inflammatory mediators. The loss of a fatty acyl chain by phospholipases from PC and PE generates lysophosphatidylcholine (LPC) and lysophophatidylethanolamine (LPE), respectively. Fatty acids can then be re-esterified to lysophospholipids by lysophospholipid acyltransferases to regenerate PC and PE, completing what is referred to as the Lands cycle [21].

In neural cell culture, HTT interacts with acidic phospholipids PI and PS [16]. However, mHTT shows an altered preference for PE over PI, and interacts with a broader array of phospholipid species compared to HTT [17]. Although mHTT interacts primarily with zwitterionic phospholipid classes PC and PE, it is the interactions of mHTT with acidic phospholipids PS and PG that result in fibrillar and oligomer aggregation in the cell [14]. In addition, the presence of the polyglutamine expansion in HTT changes its distribution within the lipid membrane, impeding the cell’s ability to clear it from the membrane [16,17]. Furthermore, the polyglutamine length influences mHTT’s interactions with phospholipids. More extended polyglutamine mutations result in more pronounced disturbances and increased disorganization of lipid bilayers in cell cultures [15]. 

The altered interaction of phospholipids with mHTT in the brain of people with HD may have profound consequences for these processes and the function of the cell membrane and its constituents (i.e., neurotransmitters, proteins). Whilst research in human post-mortem brain tissue is limited, there is evidence for region-specific decreases in PE between striatal subregions in HD (HD cases, *n* = 8), the caudate and putamen; the caudate showing more substantial losses [22]. The subventricular zone, which borders the caudate, has abnormal concentrations and distribution of multiple phospholipid classes in post-mortem human brain tissue (HD cases, *n* = 4) [23]. However, a considerable portion of knowledge concerning how and where phospholipids are altered in the human HD brain is absent. 

The following study was designed to characterize the phospholipid profiles of multiple brain regions in HD post-mortem tissue and compare these to age- and sex-matched control tissue. The selected brain regions include those that are most severely degenerated in HD (caudate, putamen), those which are moderately degenerated (grey and white matter of the cortex) and those which are mildly degenerated (cerebellum). The use of these brain regions was to aid in understanding if phospholipid disturbance is region-specific in advanced HD. It appears that in clinically advanced HD cases, phospholipid disturbance is specific to brain regions. 

## 2. Materials and Methods

### 2.1. Human Brain Tissue 

The Victorian Brain Bank supplied human post-mortem brain tissue from 13 advanced-stage HD subjects and 13 age- and sex-matched controls. Subject demographics have been published previously [24]. The Victorian Clinical Genetics Service determined the CAG repeat length of HD subjects’ disease causing HTT gene. Tissue was taken from the brain’s left hemisphere from five regions of interest: caudate, putamen, cerebellum, and the grey and white matter of the dorsomedial prefrontal cortex. The mean age, post-mortem interval, and brain pH between HD and controls were not different as determined by Kreilaus et al., 2016 [25]. All HD subjects had a Vonsattel pathological grading of IV [26] and were of advanced clinical stage. Brain tissue was stored at −80 °C until use. Ethics approval was granted by the UOW Human Research Ethics Committee (10/327), and this research was carried out in accordance with the Declaration of Helsinki (2008). 

### 2.2. Lipid Extraction 

Lipids were extracted from the human brain tissue using a modified methyl tert-butyl ether (MTBE) method described previously [27,28]. Brain tissue (~10 mg) was homogenized in 300 µL of methanol (MeOH; LC-MS Grade, VWR International, Tingalpa, QLD, Australia) containing 0.01% butylated hydroxytoluene (Sigma Aldrich, North Ryde, NSW, Australia) and internal standards (1 nmol LPC 14:0, 1 nmol LPE 14:0, 40 nmol PC 19:0/19:0, 40 nmol PE 17:0/17:0, 5 nmol PS 17:0/17:0; Avanti Polar Lipids Inc., Alabaster, AL, USA) using 1.4 mm ceramic beads in a FastPrep24 homogenizer at 6 m/s for 40 s. The homogenate was transferred to a 2 mL glass vial, and the beads washed with 100 µL MeOH. MTBE (920 µL; HPLC Grade, Bio-Strategy, Murarrie, NSW, Australia) was added to each sample, then rotated for 1 h at room temperature (25 °C). Ammonium acetate (230 µL of 150 mM; HPLC Grade, Sigma Aldrich, North Ryde, NSW, Australia) was added to each sample before being vortexed and centrifuged at 2000× *g* for 5 min. The top organic phase was removed from each sample and transferred to a new 2 mL glass vial for storage at −20 °C. Extracts were diluted 500-fold in a 2:1 MeOH: chloroform (*v*/*v*) solution with 5 mM ammonium acetate for mass spectrometric analysis. 

### 2.3. Mass Spectrometry 

Nanoelectrospray ionization mass spectrometry of lipid extracts was performed using a hybrid triple quadrupole linear ion trap mass spectrometer (Q-Trap 5500, Sciex, Vaughan, ON, Canada), equipped with an automated chip-based nanoelectrospray source (TriVersa Nanomate, Advion Biosciences, Ithaca, NY, USA) as described previously [29]. Samples were loaded into a 96-well plate and sealed before direct infusion. Spray parameters were set at a gas pressure of 0.4 psi and a voltage of 1.2 kV for positive and 1.1 kV for the negative ion mode. Lipid data were acquired using targeted precursor ion scans, as shown in Supplementary Tables S1 and S2. Target lists for each molecular species were generated following a manual review of spectra in Analyst (v1.6; Sciex, Framingham, MA, USA). The mass spectrometry output was then analyzed using LipidView (v1.2; Sciex, Framingham, MA, USA) and quantified by comparing peak areas to class-specific internal standards. Processing settings were set at a mass tolerance of 0.5 kDa and a minimum signal to noise of 20. Smoothing and de-isotoping were enabled. 

### 2.4. Data Processing

LipidView output was exported to Microsoft Excel. Positive ion data for phospholipid head groups were used to quantify lipids by matching the detected rations of isobaric species from the paired negative ion data for the fatty acids. Quantification of ether-linked phospholipid species was corrected using a 3.45 isotope correction factor as described previously [28]. Any lipids detected in ‘blank’ samples were subtracted from patient samples. Lipid species that were not detected in at least 60% of samples were excluded from the analysis. 

### 2.5. Statistical Analysis

Outliers were identified using the 2.2 interquartile range of each phospholipid class total. Lipids were assessed individually for normality using the D’Agostino–Pearson Omnibus test and then analyzed using either a two-tailed unpaired *t*-test with Welch’s correction or the Mann–Whitney U test where appropriate. Correlation analyses were conducted using Pearson’s correlation. A two-way ANOVA was used to assess differences in lipid class totals between regions in control and HD subjects. Data were adjusted for a False Discovery Rate of 1% using the two-stage Benjamini, Krieger, and Yekutieli method for multiple comparisons. Statistical test information is provided for each lipid species in Supplementary Tables S3–S68. Principal component analyses were used to compare phospholipid composition between regions in control and HD subjects. Volcano plots were created manually to visualize patterns of lipid changes between HD and controls. Data are expressed as the mean ± the standard error of the mean in nmol lipid per mg brain tissue. Processed lipid values are available in Supplementary Excel File.

## 3. Results

### 3.1. Principal Component Analyses of Control and HD Brain Regions 

**Figure 1:**
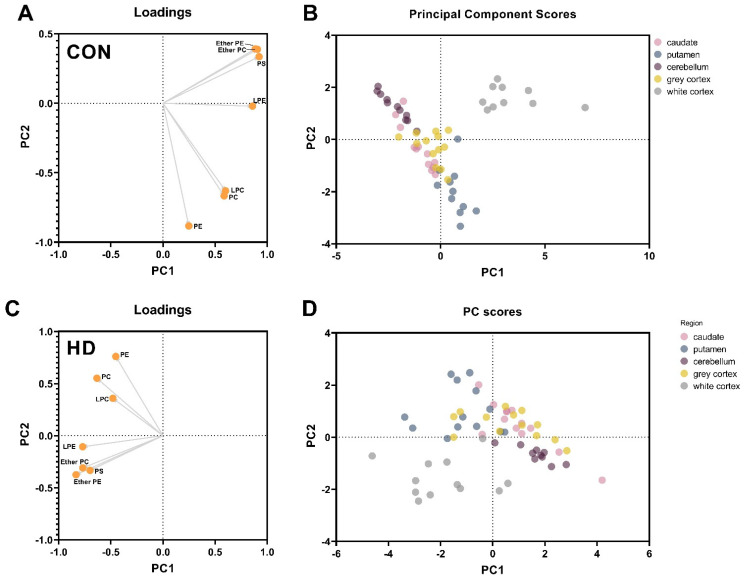
Principal Component Analysis of Phospholipids in Control and HD Brain Regions. The loading plots (**A**,**C**) demonstrate the components (phospholipid classes) which are primarily responsible for the separation of regions in control subjects (**B**) and HD subjects (**D**). Abbreviations: CON, control; HD, Huntington’s disease; PC1/PC2, principal component 1/2; LPC, lysophosphatidylcholine; LPE, lysophophatidylethanolamine; PC, phosphatidylcholine; PE, phosphatidylethanolamine; PS, phosphatidylserine.

Principal component analyses were used to determine the phospholipid classes which contributed to the separation of brain regions according to their phospholipid chemistry in control and HD subjects (Figure 1). Variances were supported using a two-way ANOVA. The controls served as a baseline for the regional variation in phospholipid content. In controls, principal component 1 (PC1) contributed to 56% of the regional variances, while component 2 (PC2) contributed 29%. The white matter of the cortex was separated from grey matter regions by significantly higher concentrations of ether PC (+60–80%), ether PE (+60–70%) and PS (+40–70%) (Figure 1A,B; PC1). The putamen was separated from the other grey matter regions by significantly higher PC (+50% caudate; +63% cerebellum), LPC (+8% caudate; 84% cerebellum) and PE (+35% caudate; +53% cerebellum). 

In comparison, the white cortex and putamen were not as clearly separated from the other regions in HD as compared to controls. HD subjects had more significant inter-subject variability in phospholipid content than controls, and therefore regions were not as well defined. In HD, PC1 contributed to 46% of the variance between regions and PC2, contributed 20%. In HD, the white matter was still distinguishable from the other grey matter regions, although not as robustly, by significantly higher concentrations of ether PC (+40–60%), ether PE (+40–60%) and PS (+40–70%) (Figure 1C,D). In HD, the putamen was separated from the other grey matter regions by higher PC (+50–60%) and PE (+30–50%), which aligned with the control separations previously mentioned. Phospholipid class totals are provided in Table 1.

### 3.2. Phosphatidylcholines

**Figure 2:**
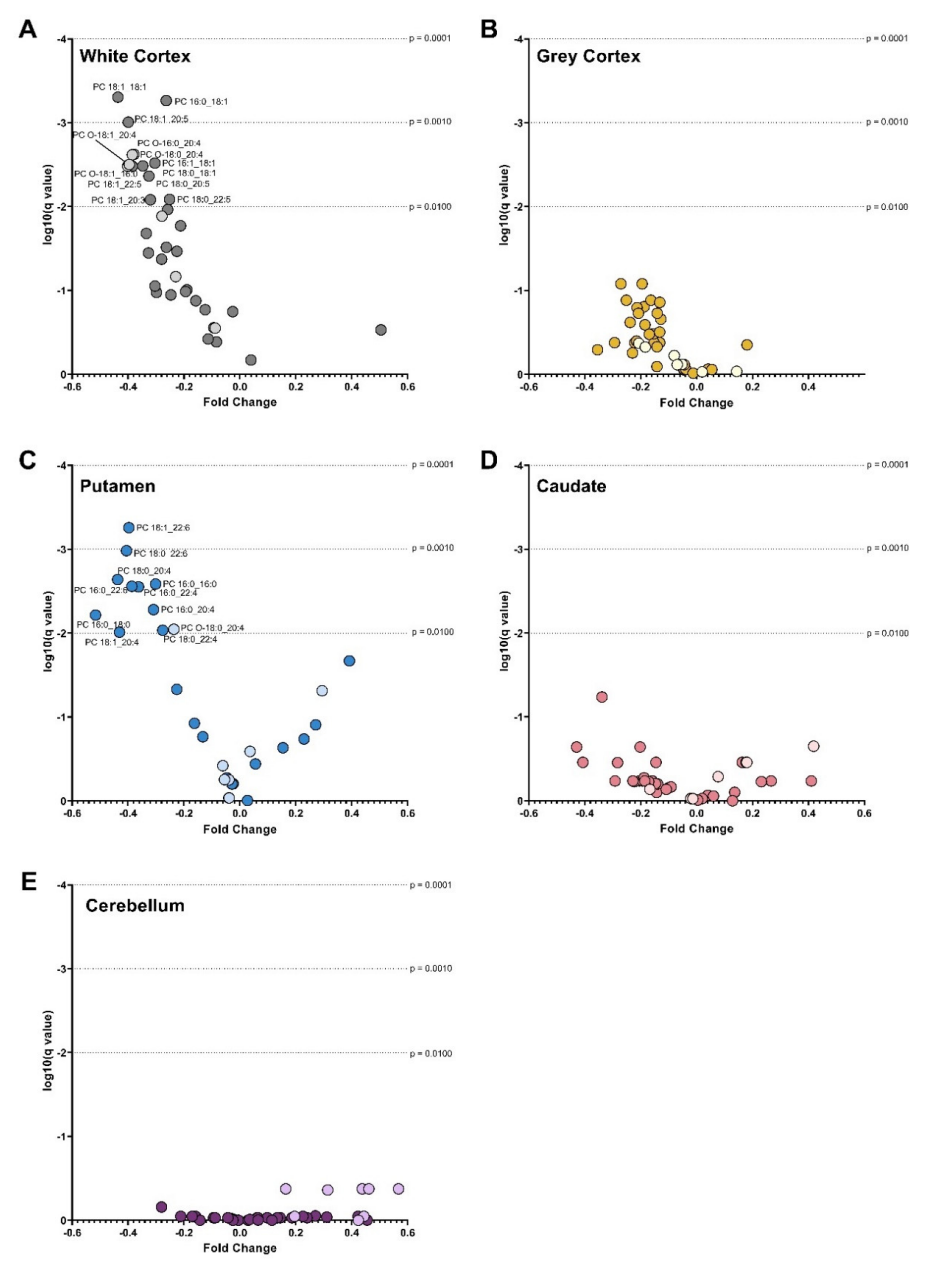
Volcano Plots of PC Species in (**A**) White Cortex, (**B**) Grey Cortex, (**C**) Putamen, (**D**) Caudate and (**E**) Cerebellum. Darker dots represent ether-linked species and lighter dots ester-linked species. The fold change between control and Huntington’s disease values is plotted on the x-axis. The log of the q values (adjusted *p* value for multiple comparisons) is plotted on the y-axis. Data were assessed for normality using the D’Agostino–Pearson Omnibus test. Depending on normality, data were either analyzed using an unpaired *t*-test with Welch’s correction or the Mann–Whitney U test. A False Discovery Rate of 1% was used to adjust for multiple comparisons (Benjamini, Krieger and Yekutieli method). Dotted lines represented alpha levels. Abbreviations: PC, phosphatidylcholine.

**Figure 3:**
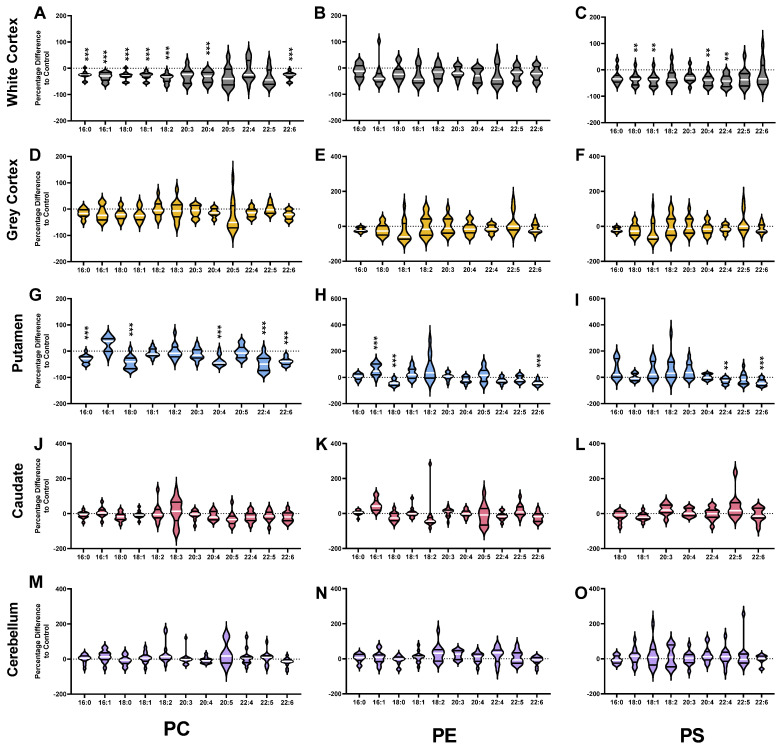
Percentage Differences of Ester-Linked PC (**A**,**D**,**G**,**J**,**M**), PE (**B**,**E**,**H**,**K**,**N**) and PS (**C**,**F**,**I**,**L**,**O**) Fatty Acids in HD Patients compared to Controls. Brain region names appear on the left-hand side of the corresponding row of graphs. Corresponding lipid classes appear at the bottom of each column of corresponding graphs. Differences were calculated using the following equation: $\left(\frac{\mathrm{HD}-\mathrm{Control}}{\mathrm{Control}}\right)\times 100\%$. Violin plots display the range, median (white line) and first and third quartiles (black lines). Data were assessed for normality using the D’Agostino–Pearson Omnibus test and analyzed using an unpaired *t*-test with Welch’s correction or the Mann–Whitney U test where appropriate. *p* values have been adjusted for a False Discovery Rate of 1% (Benjamini, Krieger and Yekutieli method). Exact *p* values are provided in the supplementary material. ** *p* < 0.01, *** *p* < 0.001. Abbreviations: PC, phosphatidylcholine; PE, phosphatidylethanolamine; PS, phosphatidylserine.

**Figure 4:**
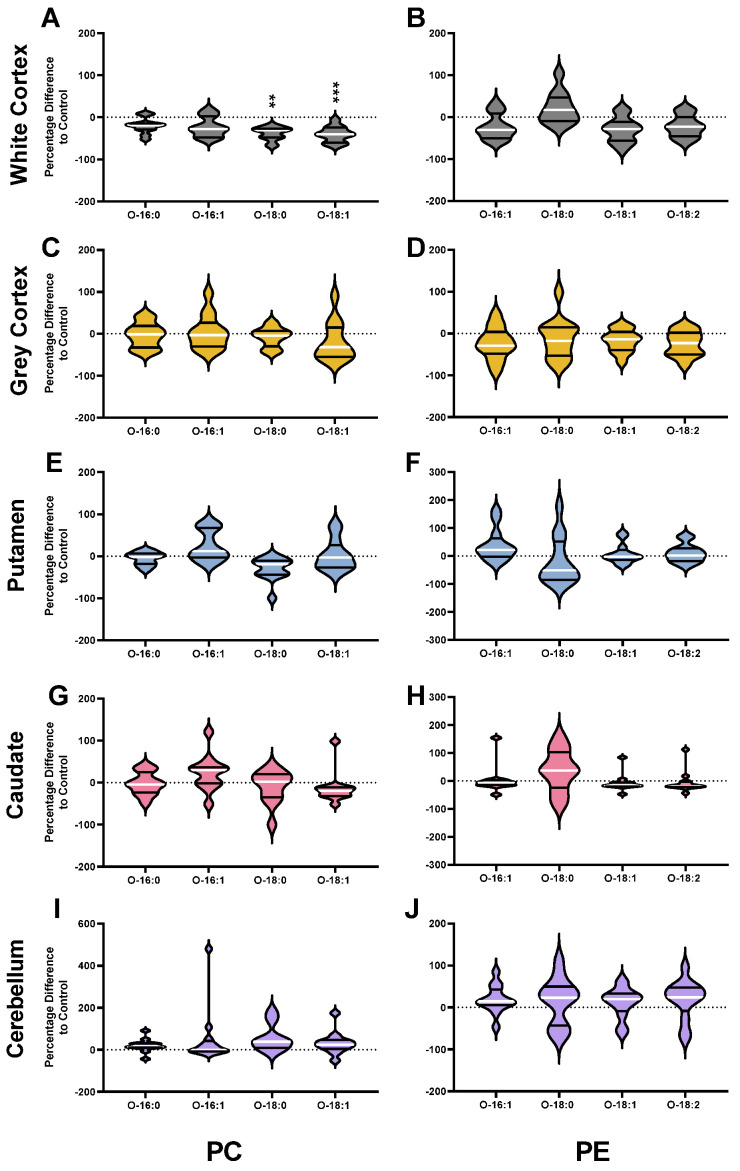
Percentage Differences of Ether-Linked PC (**A**,**C**,**E**,**G**,**I**) and PE (**B**,**D**,**F**,**H**,**J**) Fatty Acids in HD Patients compared to Controls. Brain region names appear on the left-hand side of the corresponding row of graphs. Corresponding lipid classes appear at the bottom of each column of corresponding graphs. Differences were calculated using the following equation: $\left(\frac{\mathrm{HD}-\mathrm{Control}}{\mathrm{Control}}\right)\times 100\%$. Violin plots are used and provide the range, median (white line) and first and third quartiles (black lines). Data were assessed for normality using the D’Agostino–Pearson Omnibus test and analyzed using an unpaired *t*-test with Welch’s correction or the Mann–Whitney U test where appropriate. *p* values have been adjusted for a False Discovery Rate of 1% (Benjamini, Krieger and Yekutieli method). Exact *p* values are provided in the Supplementary Material. ** *p* < 0.01, *** *p* < 0.001. Abbreviations: PC, phosphatidylcholine; PE, phosphatidylethanolamine.

**Figure 5:**
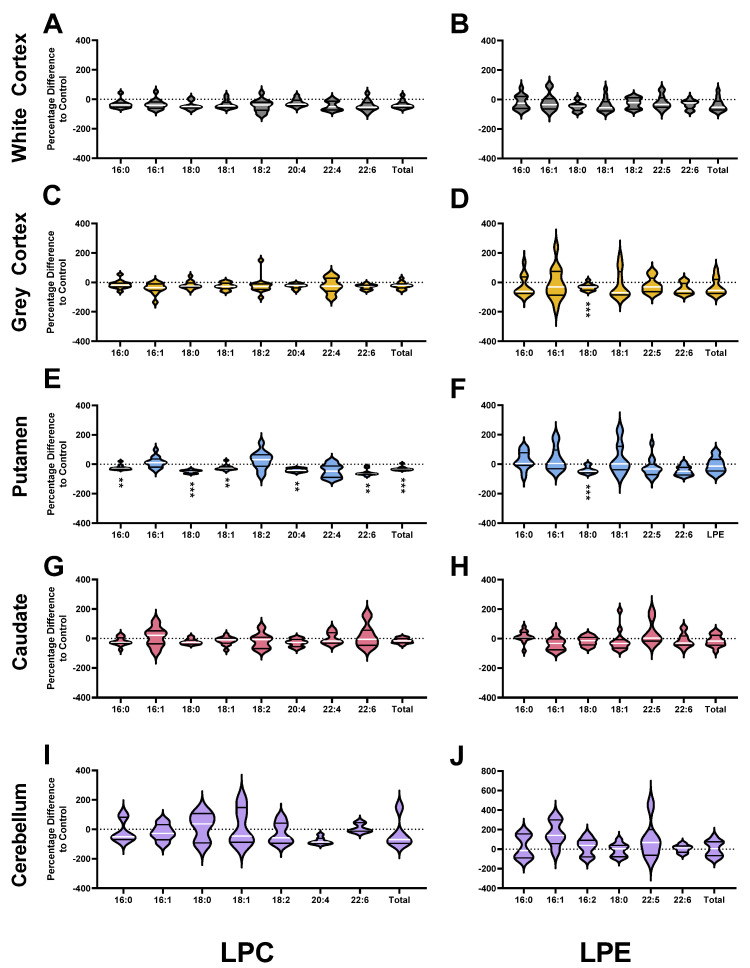
Percentage Differences of LPC (**A**,**C**,**E**,**G**,**I**) and LPE (**B**,**D**,**F**,**H**,**J**) species in HD Patients compared to Controls. Brain region names appear on the left-hand side of the corresponding row of graphs. Corresponding lipid classes appear at the bottom of each column of corresponding graphs. Differences were calculated using the following equation: $\left(\frac{\mathrm{HD}-\mathrm{Control}}{\mathrm{Control}}\right)\times 100\%$. Violin plots are used and provide the range, median (white line) and first and third quartiles (black lines). Data were assessed for normality using the D’Agostino–Pearson Omnibus test and analyzed using an unpaired *t*-test with Welch’s correction or the Mann–Whitney U test where appropriate. *p* values have been adjusted for a False Discovery Rate of 1% (Benjamini, Krieger and Yekutieli method). Exact *p* values are provided in the Supplementary Material. ** *p* < 0.01, *** *p* < 0.001. Abbreviations: LPC, lysophosphatidylcholine; LPE, lysophophatidylethanolamine.

HD patients had 28% less PC (*p* < 0.0001) in the white matter of the cortex compared with controls. The lower PC in HD was the result of a 25–40% lower abundance of several ester-linked species: PC 16:0_18:1 (*p* = 0.0002), PC 16:1_18:1 (*p* = 0.0002), PC 18:0_18:1 (*p* = 0.0005), PC 18:1_18:1 (*p* < 0.0001), PC 18:1_20:3 (*p* = 0.0029), PC 18:0_20:5 (*p* = 0.0010), PC 18:1_20:5 (*p* = 0.0001), PC 18:0_22:5 (*p* = 0.0047) and PC 18:1_22:5 (*p* = 0.0007) (Figure 2A). In the white cortex of HD, several ether-linked species were lower by approximately 40%: PC O-18:1_16:0 (*p* = 0.0007), PC O-16:0_20:4 (*p* = 0.0010), PC O-18:0_20:4 (*p* = 0.0012) and PC O-18:1_20:4 (*p* = 0.0004). The significant reductions in ester-linked 16:0 (−27%, *p* = 0.0002), 16:1 (−33%, *p* = 0.0005), 18:0 (−29%, *p* = 0.0005), 18:1 (−29%, *p* = 0.0001), 18:2 (−39%, *p* < 0.0001), 20:4 (−34%, *p* = 0.0007) and 22:6 (−29%, *p* = 0.0004) in HD patients reflect this (Figure 3A). The white cortex was the only brain region to have decreases in ether-linked PC-derived fatty acids. PC-derived O-18:0 was decreased by 39% (*p* = 0.0012) and O-18:1 by 40% (*p* = 0.0006) in HD compared to controls (Figure 4A). LPC species were not different in HD patients in the white cortex (Figure 5A). 

Compared with controls, HD patients had 28% less PC (*p* < 0.0001) in the putamen. The lower PC was due to significant reductions in PC species containing polyunsaturated fatty acyl chains: PC 16:0_22:4 (−36%, *p* = 0.0007), PC 18:0_20:4 (−44%, *p* = 0.0004), PC 16:0_20:4 (−31%, *p* = 0.0015), PC 18:1_20:4 (−43%, *p* = 0.0036), PC 16:0_22:6 (−39%, *p* = 0.0006), PC 18:0_22:4 (−28%, *p* = 0.0033), PC 18:0_22:6 (−41%, *p* = 0.0001), PC 18:1_22:6 (−40%, *p* < 0.0001) PC 16:0_16:0 (−30%, *p* = 0.0005), and PC 16:0_18:0 (−52%, *p* = 0.0020) (Figure 2C). 

Due to this, ester-linked PC-derived 16:0 (−30%, *p* = 0.0001), 18:0 (−44%, *p* = 0.0005), 20:4 (−37%, *p* = 0.0005), 22:4 (−47%, *p* = 0.0001) and 22:6 (−40%, *p* = 0.0001) was lower in HD patients (Figure 3G). In addition, multiple LPC species were lower in the putamen of HD patients. The most significant differences in LPC species identified in the putamen were in LPC 18:0 (−49%, *p* < 0.0001) and LPC 22:6 (−59%, *p* = 0.0008) (Figure 5E). The remaining species were LPC 16:0 (−24%, *p* = 0.0012), LPC 18:1 (−23%, *p* = 0.0022) and LPC 20:4 (−40%, *p* = 0.0010). No differences in PC, ether PC nor LPC were found between HD and control in the caudate (Figure 2D), or cerebellum (Figure 2E). Total PC was lower in the grey cortex of HD subjects (*p* < 0.0001); however, no changes in ether PC or LPC were found (Figure 2B). Statistical test information for PC, ether PC and LPC can be found in Supplementary Tables S3–S6 and S15 (caudate), Supplementary Tables S16–S19 and S29 (putamen), Supplementary Tables S30–S33 and S42 (white cortex), Supplementary Tables S43–46 and S55 (grey cortex) and Supplementary Tables S56–S59 and S68 (cerebellum).

### 3.3. Phosphatidylethanolamines

**Figure 6:**
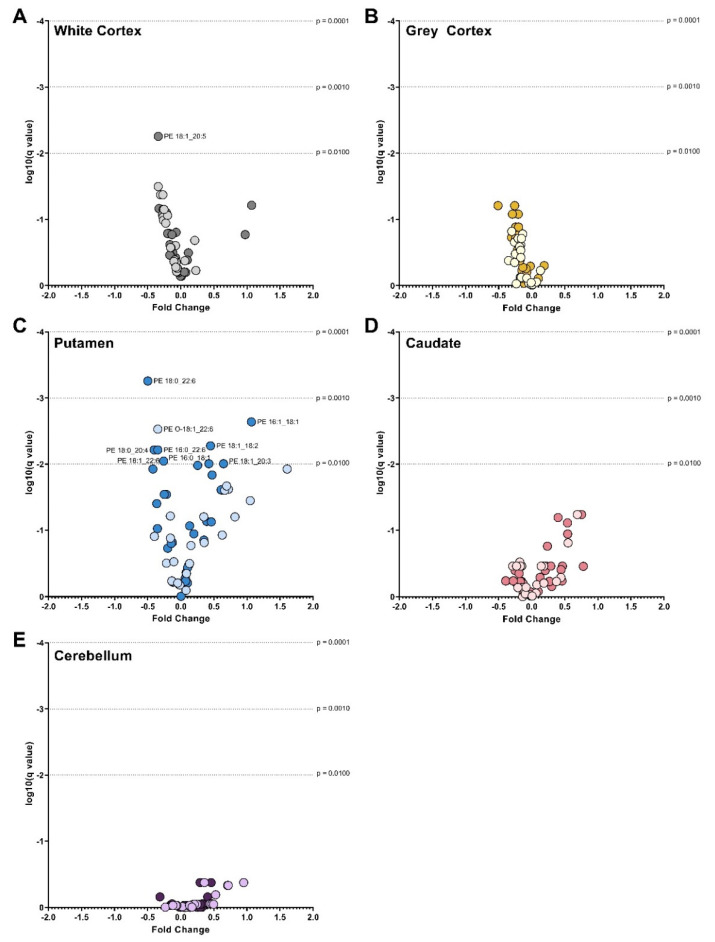
Volcano Plots of PE Species in (**A**) White Cortex, (**B**) Grey Cortex, (**C**) Putamen, (**D**) Caudate and (**E**) Cerebellum. Darker dots represent ether-linked species and lighter dots ester-linked species. The fold change between control and Huntington’s disease values is plotted on the x-axis. The log of the q values (adjusted *p* value for multiple comparisons) is plotted on the y-axis. Data were assessed for normality using the D’Agostino–Pearson Omnibus test. Data were either analyzed using an unpaired *t*-test with Welch’s correction or the Mann–Whitney U test depending on normality. A False Discovery Rate of 1% was adjusted for multiple comparisons (Benjamini, Krieger and Yekutieli method). Dotted lines represented alpha levels. Abbreviations: PE, phosphatidylethanolamine.

Ether PE was not different in any HD brain region when compared to controls. PE was lower in the putamen of HD subjects compared to controls (*p* < 0.0001). Lower PE in HD putamen was due to significant alterations in ester PE species. These shifts also caused a shifted dominance of long-chain over very long fatty acyl chain species. PE species enriched in 22:6 were lower in the putamen of HD patients: PE O-18:1_22:6 (−35%, *p* = 0.0007), PE 18:1_22:6 (−26%, *p* = 0.0031), PE 18:0_22:6 (−50%, *p* < 0.0001), and PE 16:0_22:6 (−35%, *p* = 0.0019) (Figure 6C). 

Ester-linked 22:6 was 42% lower in HD patients (*p* < 0.0001; Figure 3H), and this was confirmed to be predominately influenced by PE and not ether PE species (−42% vs. −13%) (Table S28). The 42% reduction in ester-linked 18:0 (*p* = 0.0002) was likely the result of the 40% reduction in PE 18:0_20:4 (*p* = 0.0020) in HD. Several PE species were increased in the putamen of HD subjects: PE 18:1_18:2 (+45%, *p* = 0.0015), PE 16:1_18:1 (+107%, *p* = 0.0003), PE 18:1_20:3 (+65%, *p* = 0.0039) and PE 16:0_18:1 (+42%, *p* = 0.0038) (Figure 6C). The 107% increase in PE 16:1_18:1 was likely influencing the detected 60% increase in ester-linked 16:1 in HD putamen (*p* = 0.0004) (Figure 3H). The species profile of PE was shifted in the putamen of HD patients, containing a higher proportion of monounsaturated fatty acids (+68%, *p* = 0.0001) and a lower proportion of saturated (−18%, *p* = 0.0001) and polyunsaturated fatty acids (−4%, *p* = 0.0015). 

Total LPE was not different in the HD brain. However, LPE 18:0 was reduced in both the grey cortex (−33%, *p* = 0.0003) (Figure 5F) and putamen (−41%, *p* = 0.0003) (Figure 5D) of HD patients.

Only one ether PE species was found to be different in the HD brain. PE O-18:1_22:6 was 35% lower in HD putamen compared to controls (*p* = 0.0007). PE species were not different in the caudate (Figure 6D), cerebellum (Figure 6E), or white or grey cortex (Figure 6B) of HD patients. Statistical test information for PE, ether PE and LPE can be found in Supplementary Tables S7–S10 and S15 (caudate), Supplementary Tables S20–S23 and S29 (putamen), Supplementary Tables S34–S37 and S42 (white cortex), Supplementary Tables S47–S50 and S55 (grey cortex) and Supplementary Tables S60–S63 and S68 (cerebellum).

### 3.4. Phosphatidylserines

**Figure 7:**
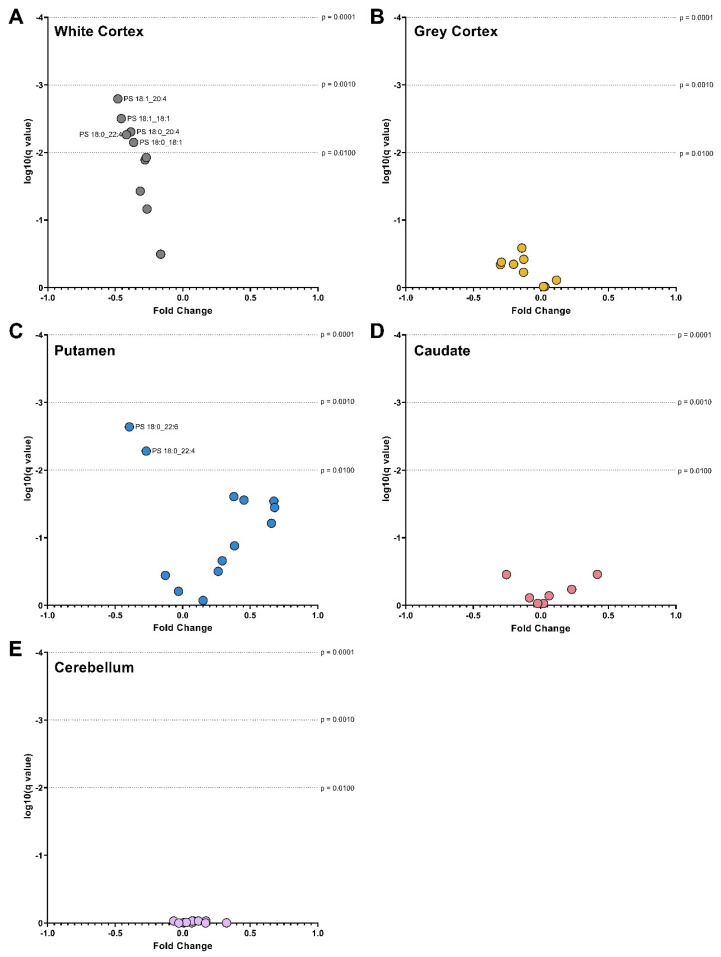
Volcano Plots of PS Species in (**A**) White Cortex, (**B**) Grey Cortex, (**C**) Putamen, (**D**) Caudate and (**E**) Cerebellum. The fold change between control and Huntington’s disease values is plotted on the x-axis. The log of the q values (adjusted *p*-value for multiple comparisons) is plotted on the y-axis. Data were assessed for normality using the D’Agostino–Pearson Omnibus test. Data were either analyzed using an unpaired *t*-test with Welch’s correction or the Mann–Whitney U test depending on normality. A False Discovery Rate of 1% was adjusted for multiple comparisons (Benjamini, Krieger and Yekutieli method). Dotted lines represented alpha levels. Abbreviations: PS, phosphatidylserine.

The white cortex was the only brain region with differences in the overall PS concentration of HD patients. PS was 37% lower in HD patients (*p* < 0.0001) due to decreases in several highly abundant species (Figure 7). PS 18:0_18:1, the most abundant species in both control and HD white cortex, was 37% lower in HD (14,390 vs. 9143 pmol/mg, *p* = 0.0024). PS 18:1_18:1, the second most abundant species, was 46% lower in HD white cortex (*p* = 0.0003). Several additional species containing polyunsaturated fatty acyl chains were also decreased in HD patients: PS 18:0_20:4 (−39%, *p* = 0.0014), PS 18:1_20:4 (−48%, *p* = 0.0007), and PS 18:0_22:4 (−42%, *p* = 0.0017) (Figure 7A). Consequently, in HD patients, PS-derived 18:0 (−35%, *p* = 0.0021), 18:1 (−39%, *p* = 0.0012), 20:4 (−40%, *p* = 0.0012) and 22:4 (−42%, *p* = 0.0017) were lower in the white cortex compared to controls (Figure 3C). 

In the putamen, HD patients had a 40% reduction in PS 18:0_22:6 (*p* = 0.0003) and a 27% reduction in PS 18:0_22:4 (*p* = 0.0014) compared with controls (Figure 7C). This aligned with proportional reductions in PS-derived 22:6 (−39%, *p* = 0.0004) and 22:4 (−27%, *p* = 0.0014) (Figure 3I). 

No changes in PS species or-derived fatty acids were identified in the caudate (Figure 3L and Figure 7D), cerebellum (Figure 3O and Figure 7E) and grey cortex (Figure 3F and Figure 7B) between control and HD patients. Statistical test information for PS can be found in Supplementary Tables S11 and S12 (caudate), Supplementary Tables S24 and S25 (putamen), Supplementary Tables S38 and S39 (white cortex), Supplementary Tables S51 and S52 (grey cortex) and Supplementary Tables S64 and S65 (cerebellum).

### 3.5. CAG Repeat Length Is Not Related to Neural Phospholipid Abundances in HD Patients

Pearson’s correlation analysis was run to determine if a relationship existed between the CAG repeat length of HD patients and the concentration of phospholipids in each brain region. The *p* values were adjusted for multiple comparisons using the two-stage Benjamini, Krieger and Yekutieli method. No significant correlations were identified between CAG repeat length and any phospholipid class, species, or fatty acyl chains. 

## 4. Discussion

The putamen and the white matter of the dorsomedial prefrontal cortex have a region-specific vulnerability to phospholipid disturbance in cases of clinically advanced HD. The vulnerability is supported by the significant changes to the phospholipid profiles of these regions and the absence of change in the caudate, cerebellum, and grey matter of the dorsomedial prefrontal cortex. Previous reports on lipid metabolism in the cerebellum of HD post-mortem tissue have found little to no changes in this brain region [25,30]. However, the caudate is a region where multiple lipid classes are altered in HD [25,30,31]. The selective reduction in the PC and PS content of the white matter in the cortex and ester-linked 22:6 in the putamen suggests that phospholipid disturbance is both region and lipid specific in advanced HD. The absence of change in ether PE in the HD brain was perplexing, considering the immense importance of these lipids to neural function and the severe degeneration of the brain in HD. Whilst the extraction and mass spectrometric analysis used in this study cannot isolate the cellular location of the identified lipid changes (i.e., neural bodies, glia, synapses), phospholipids are most abundant in the cell membrane contributing to fluidity and function. Thus, this study revealed a critical feature of HD; not all brain regions experience the same disturbance in phospholipid metabolism. The nature of the changes to phospholipids between the caudate and putamen suggest that they are specific not only to whole regions, but also striatal subregions. In controls, the caudate and putamen were separated by PC and PE composition, as shown in the principal component analysis (Figure 1A,B). Considering the caudate and putamen share the same dominant cell type (~95% medium spiny neurons), their distinct phospholipid profiles were interesting. The putamen had almost twice the PC content compared with the caudate, and PC was the phospholipid which was reduced by 28% in the putamen of HD patients. The PE content of the putamen in controls was also significantly higher than the caudate, and again only the putamen was affected by changes in PE composition in HD. The difference in the phospholipid chemistry between the caudate and putamen in controls may simply be the result of changes in the numbers of neural cells (i.e., astrocytes, microglia), an infiltration of the biochemistry of neighboring brain regions (i.e., internal capsule), or it may reflect fundamental biochemical differences between striatal subregions. The difference in phospholipid disturbance between the caudate and putamen in HD may be explained by the differing rates and onset of degeneration, as well as their differing relationships to clinical indices in HD [32,33]. However, the previous investigation into cholesterol metabolism in HD has also indicated a difference in the vulnerability to lipid disturbance between the caudate and putamen [25,31]. The selective disturbance of PC and PE in the putamen of HD patients may reflect a difference in the molecular consequences or response to pathological triggers caused by HD compared to the caudate. The shift in the relative abundance of PE species towards an increased dominance of monounsaturated fatty acyl species in the putamen suggests alterations in cell membrane fluidity and permeability in HD, affecting cell function. The abundance of ester-linked 22:6 (also known as docosahexaenoic acid; DHA)-derived from PC, LPC, PE, and PS was lower in the putamen of HD patients. These reductions were specific to the putamen; no reductions in polyunsaturated fatty acid chains were identified in the caudate, which houses the same neuronal population as the putamen (medium spiny neurons). Mammals cannot synthesize 22:6, so the brain contributes to the abundance of 22:6 via the elongation and desaturation of dietary α-linolenic acid [34], the final step of which occurs exclusively in astrocytes [35]. Astrocytes can then mediate the release of 22:6 for incorporation into phospholipids [36] and inflammatory mediators maresins, resolvins and protectins [37,38]. Severe astrocytosis (an increase in the number of astrocytes) in the caudate and putamen indicates pathological grade IV classification of HD post-mortem tissue used in this study [26]. Neural inflammation is well documented in HD [37,39,40]. An increase in astrocytes would suggest an increase in the available 22:6, unless it is siphoned to inflammatory mediators, restricting the availability to phospholipids. Alternative explanations include a release of 22:6 by phospholipids for inflammatory mediators or changes in the transport of 22:6 into the brain by LPC. LPC is the favoured carrier of dietary 22:6 across the blood–brain barrier [41], and the reduction in LPC and PC would support this. 

Although the exact *sn*-position of the fatty acyl chains was not determined, polyunsaturated chains (including 22:6) typically attach to phospholipids at the *sn*-2 position of the glycerol backbone. The release of fatty acyl chains at the *sn*-2 position is facilitated by phospholipase A_2_ (PLA_2_) [42]. PC, PE, and PS share synthesis and degradation pathways, making it difficult to discern if the reduction of 22:6 in one phospholipid class then had downstream effects on another. Fatty acyl chains are rapidly hydrolyzed and re-esterified to phospholipids allowing them to ‘shuffle’ between classes as needed by the cell [18]. Compared with other phospholipid classes, PS is enriched in 22:6 and thus acts as a storage facility for the fatty acid [43]. PS 18:0_22:6 is the most abundant PS species in the brain [36], and this species was the driving force of the reductions in PS-derived 22:6 in HD putamen. In neural cell cultures, 22:6 supplementation assists neurite growth and increases the number of dendritic spines, promoting more quality connections [36]. In addition, the esterification of 22:6 to PS allows the species to incur anti-apoptotic effects in vitro. These effects are thought to occur because of the ability of 22:6 PS species’ ability to promote brain-derived neurotrophic factor induced translocation of Raf-1 kinase in the cell membrane, which is crucial to cell survival [36,43]. Other fatty acids, including 18:1 and 22:5, cannot rescue this feature in cells when 22:6 is absent. Therefore, the loss of 22:6 from PS species in the putamen of people with HD could have profound consequences for cell survival.

The white matter of HD patients was distinguishable from the other brain regions, by lower abundances of PC and PS, in the dorsomedial prefrontal cortex. If these lower abundances were a direct result of neuronal degeneration or atrophy, it is expected that PE (including ether) would be decreased by a similar fold. The principal component analysis of controls (Figure 1A,B) indicated that white matter was distinguishable from the grey matter by ether PC, ether PE and PS content. The separation of matter type is likely due to the strong associations of these lipids with myelin, the dominant component of white matter. PC, PE, and PS represent 8.3%, 11.2% and 5.3% of the total lipid dry weight, respectively, in the aged brain [44]. Of PE, ether species are reported to contribute up to 70% [45]. Magnetic resonance imaging studies report decreases in the volume and structural abnormalities in major white matter tracts early in HD; however, there is little evidence of the molecular changes occurring [46,47,48]. Myelin breakdown in the white matter tracts surrounding the basal ganglia and those connecting it to the frontal cortex have been reported [47]. The high abundance of PE and ether PE in myelin and the absence of change in these lipids in the white matter of HD patients suggests that the selective reductions in PC and PS are either non-myelin related or selective targeting of these lipids within the myelin membrane.

Despite changes to multiple phospholipids in the putamen and cortical white matter of HD patients, ether PE species and ether-linked PE fatty acyl chains appear to be protected across multiple regions of HD brain. Albeit for one ether PE species in the putamen, these lipids and their ether-linked fatty acyl chains were unaltered in all five brain regions in HD. The reason for this is unclear, considering their high abundance in the brain and the enormous contribution that they have to neural function. Early reports on HD post-mortem brain tissue found no differences in the relative abundance of PE to PE plasmalogens in the caudate. However, these reports could not ascertain exact quantities [49]. The ether PE detected in this study include species with both alkyl ether bonds and vinyl ether bonds. The difference between these bonds is not easily detectable in phospholipids and the mass spectrometric techniques used could not distinguish them. Ether PE species with vinyl ether bonds are plasmalogens. There is evidence that PE plasmalogens are protective against oxidative damage. This protection is due to the ease of oxidation of their vinyl ether bond at the sn-1 position compared to the ester bond at the sn-1 position of diacyl species [50]. The evidence suggests that once vinyl ether bonds are oxidized, the propagation of reactive oxygen species, which typically occurs in ester-linked species, is stopped, effectively halting the oxidative event cascade [51]. Furthermore, incorporating highly oxidizable polyunsaturated fatty acids into ether PE is suggested to protect the fatty acids against oxidation [51].

It is established that ester-linked fatty acyl chains are cleaved from phospholipids by either phospholipase A_1_ for those at the *sn*-1 position or PLA_2_ at the *sn*-2 position. However, the ester-linked fatty acyl chains at the *sn*-2 position of ether PE are released via the selective phospholipase PlsEtn-PLA_2_ [52]. The ether-linked fatty acyl chains at the *sn*-1 position of ether PE are cleaved via oxidation. The decrease in PE species’ polyunsaturated fatty acid content in HD putamen was caused by a reduced abundance of PE species, not ether PE species. For example, the reduction in 22:6 from ester PE species was 42% (*p* = 0.001), whilst the reduction from ether PE species was 12% (*p* = 0.099). Therefore, reductions in the highly oxidative suspectable polyunsaturated fatty acids would likely be due to PLA_2_, releasing polyunsaturated fatty acyl chains from PE and not PlsEtn-PLA_2_ from ether PE. PLA_2_ is coupled to dopaminergic [53], serotonergic [54], and N-methyl _D_-aspartate [55] neuroreceptors, all affected in HD [56,57,58].

## 5. Conclusions

Changes to phospholipids are isolated to specific brain regions in advanced HD. In this study, those changes were in the white matter of the dorsomedial prefrontal cortex and the putamen. The absence of change to phospholipids in the caudate indicates that cell type may not dictate a region’s susceptibility to phospholipid disturbance in HD; it may be that location in the brain or the cellular response of a specific region to pathological stimuli. It appears that ether bonds in PE may protect attached fatty acyl chains in HD, supporting evidence from in vitro studies that the molecular structure of phospholipids can influence its susceptibility to interference by mutant huntingtin. The selective changes in phospholipid classes in the white matter of the dorsomedial prefrontal cortex and the putamen prompt vital considerations for using lipid therapeutics to treat HD, as each brain region may need unique approaches.

## Figures and Tables

**Table 1:** Phospholipid Class Totals in Brain Regions of Control and HD Subjects.

Lipid	Brain Region	CONTROL	HD	PD (%)	*p*
PC	White Cortex	25,402.41 ± 1254.05 (11)	18,182.46 ± 1155.50 (11)	−28.42	<0.0001 ***
	Grey Cortex	19,323.14 ± 1077.29 (13)	15,835.15 ± 1038.53 (13)	−18.05	<0.0001 ***
	Putamen	36,248.62 ± 1480.35 (12)	25,940.88 ± 1415.30 (13)	−28.44	<0.0001 ***
	Caudate	18,214.87 ± 1187.31 (13)	16,558.68 ± 1019.03 (12)	−9.09	0.0656
	Cerebellum	13,285.99 ± 1251.90 (12)	13,420.99 ± 902.97 (13)	1.02	0.8806
Ether PC	White Cortex	1979.21 ± 148.62 (11)	1228.02 ± 104.97 (11)	−37.95	0.4327
	Grey Cortex	561.59 ± 62.31 (13)	490.65 ± 61.45 (13)	−12.63	0.9358
	Putamen	758.35 ± 122.87 (12)	760.10 ± 59.79 (13)	0.23	0.9984
	Caudate	545.78 ± 75.12 (13)	503.68 ± 50.50 (12)	−7.71	0.9626
	Cerebellum	368.99 ± 31.65 (12)	492.14 ± 44.46 (13)	33.38	0.8910
PE	White Cortex	6946.47 ± 480.46 (12)	6022.46 ± 596.55 (12)	−13.3	0.3135
	Grey Cortex	10,430.49 ± 491.76 (13)	8081.10 ± 489.24 (13)	−22.52	0.0078 *^ns^*
	Putamen	12,304.40 ± 670.80 (12)	8771.73 ± 673.31 (13)	−28.71	<0.0001 ***
	Caudate	8034.76 ± 837.44 (13)	7050.75 ± 476.51 (12)	−12.25	0.2737
	Cerebellum	5829.33 ± 612.97 (12)	5625.40 ± 413.87 (13)	−3.5	0.8205
Ether PE	White Cortex	14,046.65 ± 1311.70 (12)	10,273.73 ± 1127.64 (12)	−26.86	<0.0001 ***
	Grey Cortex	5382.45 ± 467.61 (13)	4303.00 ± 427.34 (13)	−20.06	0.2204
	Putamen	5678.41 ± 324.75 (12)	6363.84 ± 573.87 (13)	12.07	0.4457
	Caudate	4080.83 ± 535.07 (13)	3889.34 ± 416.19 (12)	−4.69	0.8313
	Cerebellum	4207.76 ± 463.00 (12)	4848.44 ± 426.84 (13)	15.23	0.4760
PS	White Cortex	20,523.65 ± 1554.27 (12)	13,021.57 ± 1375.81 (12)	−36.55	<0.0001 ***
	Grey Cortex	7393.91 ± 747.16 (13)	5871.27 ± 711.59 (13)	−20.59	0.0841
	Putamen	7645.28 ± 378.45 (12)	7935.62 ± 626.60 (13)	3.8	0.7466
	Caudate	5106.90 ± 339.73 (12)	4502.53 ± 407.56 (12)	−11.83	0.5097
	Cerebellum	3542.65 ± 406.07 (12)	3916.13 ± 427.30 (13)	10.54	0.6777
LPC	White Cortex	124.85 ± 12.68 (12)	79.38 ± 9.14 (13)	−36.41	0.9597
	Grey Cortex	100.86 ± 4.71 (13)	78.88 ± 6.40 (13)	−21.78	0.9801
	Putamen	144.80 ± 8.99 (13)	100.77 ± 5.05 (13)	−30.41	0.9601
	Caudate	133.10 ± 6.65 (13)	111.74 ± 6.44 (13)	−16.05	0.9806
	Cerebellum	22.52 ± 6.75 (11)	15.95 ± 6.95 (10)	−29.16	0.9947
LPE	White Cortex	1153.67 ± 158.31 (12)	729.78 ± 138.85 (13)	−36.74	0.6372
	Grey Cortex	510.41 ± 66.15 (13)	360.13 ± 80.77 (13)	−29.44	0.8645
	Putamen	574.38 ± 67.27 (13)	541.14 ± 81.68 (13)	−5.79	0.9699
	Caudate	317.12 ± 30.20 (13)	271.58 ± 34.41 (13)	−14.36	0.9588
	Cerebellum	78.65 ± 22.48 (11)	82.90 ± 17.78 (12)	5.4	0.9964

Data are presented as the mean ± SEM (n) in pmol lipid per mg tissue. Data were assessed using a two-way ANOVA. Multiple comparisons were adjusted using a False Discovery Rate of 1% (Benjamini, Krieger and Yekutieli method). The percentage difference of HD compared to controls is provided. *** *p* < 0.001. *^ns^*, not significant when adjusted for multiple comparisons. Abbreviations: CON, control; HD, Huntington’s disease; LPC, lysophosphatidylcholine; LPE, lysophophatidylethanolamine; PC, phosphatidylcholine; PD, percentage difference; PE, phosphatidylethanolamine; PS, phosphatidylserine; SEM, standard error of the mean.

## Data Availability

Processed lipid values, and statistics can be found in the Supplementary Excel File and tabled in the Supplementary Material PDF.

## Supplementary Materials

The following supporting information can be downloaded at: https://www.mdpi.com/article/10.3390/nu14102086/s1, Supplementary Tables: Table S1, Positive precursor ion and neutral loss scans for phospholipids; Table S2, Negative Precursor Ion Scans for Phospholipid Fatty Acyl Chain Identification; Table S3, Ester PC Species in Control and HD Caudate; Table S4, Ether PC Species in Control and HD Caudate; Table S5, PC Ester Linked Fatty Acids in Control and HD Caudate; Table S6, PC Ether Linked Fatty Acids in Control and HD Caudate; Table S7, Ester PE Species in Control and HD Caudate; Table S8, Ether PE Species in Control and HD Caudate; Table S9, PE Ester Linked Fatty Acids in Control and HD Caudate; Table S10, PE Ether Linked Fatty Acids in Control and HD Caudate; Table S11, PS Species in Control and HD Caudate; Table S12, PS Derived Fatty Acids in Control and HD Caudate; Table S13, Total Ester Linked Phospholipid Derived Fatty Acids in Control and HD Caudate; Table S14, Phospholipid Class Totals in Control and HD Caudate; Table S15, LPC and LPE species in control and HD caudate; Table S16, Ester PC Species in Control and HD Putamen; Table S17, Ether PC Species in Control and HD Putamen; Table S18, PC Ester Linked Fatty Acids in Control and HD Putamen; Table S19, PC Ether Linked Fatty Acids in Control and HD Putamen; Table S20, Ester PE Species in Control and HD Putamen; Table S21, Ether PE Species in Control and HD Putamen; Table S22, PE Ester Linked Fatty Acids in Control and HD Putamen; Table S23, PE Ether Linked Fatty Acids in Control and HD Putamen; Table S24, PS Species in Control and HD Putamen; Table S25, PS Fatty Acids in Control and HD Putamen; Table S26, Total Phospholipid Derived Fatty Acids in Control and HD Putamen; Table S27, Phospholipid Class Totals in Control and HD Putamen; Table S28, Confirmation Analysis of Origin of 22:6 Reductions in PE Species; Table S29, LPC and LPE species in control and HD putamen; Table S30, Ester PC Species in Control and HD White Cortex; Table S31, Ether PC Species in Control and HD White Cortex; Table S32, Ester Linked PC Fatty Acids in Control and HD White Cortex; Table S33, Ether Linked PC Fatty Acids in Control and HD White Cortex; Table S34, Ester PE Species in Control and HD White Cortex; Table S35, Ether PE Species in Control and HD White Cortex; Table S36, Ester Linked PE Fatty Acids in Control and HD White Cortex; Table S37, Ether Linked PE Fatty Acids in Control and HD White Cortex; Table S38, PS Species in Control and HD White Cortex; Table S39, PS Fatty Acids in Control and HD White Cortex; Table S40, Total Phospholipid Derived Ester Linked Fatty Acids in Control and HD White Cortex; Table S41, Phospholipid Class Totals in Control and HD White Cortex; Table S42, LPC and LPE in Control and HD White Cortex; Table S43, Ester PC Species in Control and HD Grey Cortex; Table S44, Ether PC Species Control and HD Grey Cortex; Table S45, Ester Linked PC Fatty Acids in Control and HD Grey Cortex; Table S46, Ether Linked PC Fatty Acids in Control and HD Grey Cortex; Table S47, Ester PE Species in Control and HD Grey Cortex; Table S48, Ether PE Species in Control and HD Grey Cortex; Table S49, Ester Linked PE Fatty Acids in Control and HD Grey Cortex; Table S50, Ether Linked Fatty Acids in Control and HD Grey Cortex; Table S51, PS Species in Control and HD Grey Cortex; Table S52, PS Fatty Acids in Control and HD Grey Cortex; Table S53, Total Phospholipid Derived Ester Linked Fatty Acids in Control and HD Grey Cortex; Table S54, Phospholipid Class Totals in Control and HD Grey Cortex; Table S55, LPC and LPE Species in Control and HD Grey Cortex; Table S56, Ester PC Species in Control and HD Cerebellum; Table S57, Ether PC Species in Control and HD Cerebellum; Table S58, Ester Linked PC Fatty Acids in Control and HD Cerebellum; Table S59, Ether Linked PC Fatty Acids in Control and HD Cerebellum; Table S60, Ester PE Species in Control and HD Cerebellum; Table S61, Ether PE Species in Control and HD Cerebellum; Table S62, Ester Linked PE Fatty Acids in Control and HD Cerebellum; Table S63, Ether Linked PE Fatty Acids in Control and HD Cerebellum; Table S64, PS Species in Control and HD Cerebellum; Table S65, PS Fatty Acids in Control and HD Cerebellum; Table S66, Total Ester Linked Phospholipid Derived Fatty Acids in Control and HD Cerebellum; Table S67, Phospholipid Class Totals in Control and HD Cerebellum; Table S68, LPC and LPE Species in Control and HD Cerebellum.

## Abbreviations

DHA, Docosahexaenoic Acid; HD, Huntington’s Disease; HPLC, High-Performance Liquid Chromatography; *HTT*, Huntingtin Gene; HTT, Huntingtin Protein; LC-MS, Liquid Chromatography Mass Spectrometry; LPC, Lysophosphatidylcholine; LPE, Lysophopshatidylethanolamine; mHTT, Mutant Huntingtin Protein; MTBE, Methyl Tert Butyl Ether; PA, Phosphatidic Acid; PC, Phosphatidylcholine; PC1/2, Principal Component 1/2; PE, Phosphatidylethanolamine; PG, Phosphatidylglycerol; PI, Phosphatidylinositol; PLA_2_, Phospholipase A_2_; PS, Phosphatidylserine.
