# Supplementary material for: Phospholipid Profiles Are Selectively Altered in the Putamen and White Frontal Cortex of Huntington’s Disease
Source: Nutrients. 2022 May 16;14(10):2086. doi: 10.3390/nu14102086 (PMC9143248; doi:10.3390/nu14102086)
Supplement: Supplementary file 1 [file nutrients-14-02086-s001.zip › Supplementary.pdf]

Supplementary Tables

Phospholipid Profiles are Selectively Altered in the Putamen and White Frontal Cortex of Huntington's Disease

Gabrielle R. Phillips[1,2,3*]; Sarah E. Hancock[4]; Andrew M. Jenner[5]; Kelly A. Newell[1,2,3]; Todd W. Mitchell[1,2,3]

AFFILIATIONS
[1]Illawarra Health and Medical Research Institute, Wollongong, 2522, NSW, Australia
[2]School of Medicine, University of Wollongong, Wollongong 2522, NSW, Australia
[3]Molecular Horizons, University of Wollongong, Wollongong 2522, NSW, Australia
[4]School of Medical Sciences, University of New South Wales, Sydney, 2052, NSW, Australia
[5]Bioanalytical Mass Spectrometry Facility, Mark Wainwright Analytical Centre, University of New South Wales, Sydney, 2052, NSW, Australia

Table S1 Positive precursor ion and neutral loss scans for phospholipids

	Scan	Mass Range	Collision Energy
**PC**	+Prec. m/z 184.1	640-850	40
**PE**	+NL 141.0	680-830	30
**PS**	+NL 185.0	730-850	30
**LPC**	+Prec. m/z 184.1	450-600	40
**LPE**	+NL 141.0	400-550	30

Abbreviations: LPC Lysophosphatidylcholine; **LPE** lysophophatidylethanolamine; **NL** Neutral Loss; **PC** Phosphatidylcholine; **PE** Phosphatidylethanolamine; **Prec.** Precursor; **PS** Phosphatidylserine.

Table S2 Negative Precursor Ion Scans for Phospholipid Fatty Acyl Chain Identification.

Fatty Acyl Chain	-Prec. Scan (m/z)	Mass Range	Collision Energy
**14:0**	227.2	580-900	-55
**16:1**	253.2	600-900	-55
**16:0**	255.2	600-900	-55
**17:0**	269.3	560-900	-55
**18:2**	279.2	600-900	-40
**18:1**	281.3	600-900	-55
**18:0**	283.3	600-900	-55
**19:0**	297.3	600-900	-55
**20:5**	301.2	500-1000	-40
**20:4**	303.2	600-1000	-40
**20:3**	305.2	600-1000	-40
**20:2**	307.2	600-1000	-40
**20:1**	309.2	600-1000	-55
**20:0**	311.2	600-1000	-55
**22:6**	327.2	700-1000	-40
**22:5**	329.2	700-1000	-40
**22:4**	331.2	700-1000	-40
**22:3**	333.3	600-1000	-40

Abbreviations: Prec. Precursor.

SUPPLEMENTARY TABLES

***For All Tables:** Data is expressed as pmol lipid per mg brain tissue. Data was assessed for normality using a D'Agostino Pearson Omnibus test and analysed using either a two-tailed unpaired t-test with Welch's correction or a Mann Whitney U test where appropriate. Data was adjusted for a False Discovery Rate of 1% using a two stage Benjamini, Krieger and Yekutieli method. Percentage differences are expressed as the difference of HD compared with controls. P values are provided with the adjusted q value for the False Discovery Rate. Only lipids labelled as a discovery were considered statistically significant.

Table S3 Ester PC Species in Control and HD Caudate.

	CON			HD						
	Mean	SEM	N	Mean	SEM	N	PD (%)	p	q	Discovery?
PC 16:0_16:0	2356.59	199.52	13	1983.45	160.00	12	24.31	0.1585	0.5797	No
PC 16:0_16:1	424.73	28.44	13	441.72	34.02	12	24.53	0.7053	0.8598	No
PC 16:0_18:0	647.40	89.27	13	458.46	60.48	12	15.54	0.0945	0.5797	No
PC 16:0_18:1	7940.22	498.13	13	7801.35	424.75	12	24.48	0.8339	0.9387	No
PC 16:1_18:0	77.46	4.37	13	82.12	10.38	12	25.44	0.8100	0.8776	No
PC 16:0_18:2	145.93	12.97	13	165.82	22.37	12	49.32	0.6495	0.7899	No
PC 16:1_18:1	155.15	11.98	13	180.45	12.68	12	38.22	0.1095	0.3481	No
PC 16:0_18:3	19.13	1.58	12	24.23	2.84	10	85.11	0.1386	0.5797	No
PC 18:0_18:1	2390.09	211.36	13	2042.80	204.59	12	12.81	0.1366	0.3481	No
PC 18:0_18:2	69.16	7.19	13	53.75	10.20	12	-24.61	0.2308	0.5911	No
PC 18:1_18:1	688.60	78.44	13	549.42	63.90	12	15.85	0.0257	0.2281	No
PC 16:0_20:3	109.61	9.48	13	98.53	11.68	12	38.13	0.4695	0.7012	No
PC 18:1_18:2	48.37	3.68	13	54.57	7.55	11	-9.97	1.0000	1.0000	No
PC 16:0_20:4	722.27	66.36	13	656.34	50.49	12	36.54	0.4376	0.6824	No
PC 16:0_20:5	18.08	2.57	13	22.26	3.06	11	54.29	0.3073	0.5911	No
PC 16:1_20:4	18.92	4.55	11	16.21	3.32	11	193.33	0.6365	0.7942	No
PC 18:0_20:3	79.37	7.96	13	80.97	4.10	12	2.56	0.8606	0.9387	No
PC 16:0_22:4	110.99	10.88	13	88.25	10.99	12	-14.38	0.1550	0.5797	No
PC 18:0_20:4	768.05	87.35	13	619.32	60.57	12	28.93	0.1763	0.5797	No
PC 18:1_20:3	42.77	7.91	13	34.73	5.63	12	60.95	0.2945	0.5319	No
PC 16:0_22:5	26.89	4.62	13	37.93	5.43	12	-10.15	0.1360	0.5797	No
PC 18:0_20:5	79.41	6.08	13	52.52	7.38	12	-40.48	0.0019	0.0579	No
PC 18:1_20:4	143.11	17.65	13	123.12	12.73	12	63.83	0.3685	0.6304	No

Table S3 Continued… Ester PC Species in Control and HD Caudate.

	CON Mean	SEM	N	HD Mean	SEM	N	PD (%)	p	q	Discovery?
**PC 16:0_22:6**	402.26	49.72	13	343.10	35.64	12	24.25	0.3443	0.6236	No
**PC 18:1_20:5**	38.06	8.57	12	21.72	6.49	11	-42.93	0.0268	0.2281	No
**PC 18:2_20:4**	4.06	1.71	10	3.62	0.73	10	-7.00	0.5159	0.7290	No
**PC 16:1_22:6**	27.88	2.06	11	27.99	1.19	10	36.69	0.9633	0.9792	No
**PC 18:0_22:4**	142.25	16.23	13	117.86	11.81	12	47.00	0.2377	0.5911	No
**PC 18:0_22:5**	76.99	6.12	13	62.72	4.38	11	1.34	0.0719	0.5797	No
**PC 18:0_22:6**	291.92	34.46	13	225.39	24.04	12	22.01	0.1282	0.5797	No
**PC 18:1_22:5**	23.49	4.64	12	13.93	2.37	10	31.61	0.1229	0.3481	No
**PC 18:1_22:6**	139.99	14.80	13	100.42	8.78	12	-12.54	0.0328	0.3506	No

Abbreviations: CON Control; **HD** Huntington's disease; **PC** Phosphatidylcholine; **PD** Percentage Difference; **PE** Phosphatidylethanolamine; **PS** Phosphatidylserine; **SEM** Standard Error of Mean.

Table S4 Ether PC Species in Control and HD Caudate.

	CON Mean	SEM	N	HD Mean	SEM	N	PD (%)	p	q	Discovery?
**PC O-16:0_16:0**	93.44	5.24	13	91.49	6.33	12	21.04	0.8149	0.9387	No
**PC O-16:1_16:0**	32.72	3.96	13	38.51	4.10	12	57.40	0.1095	0.3481	No
**PC O-18:1_16:0**	318.48	70.66	13	265.19	36.78	12	-2.64	0.5382	0.7290	No
**PC O-16:0_20:4**	26.54	2.37	13	31.32	2.14	10	69.96	0.0769	0.3481	No
**PC O-16:1_20:4**	10.06	1.04	12	14.27	1.19	11	38.96	0.0147	0.2232	No
**PC O-18:0_20:4**	37.53	2.16	13	37.04	3.13	11	28.97	0.8984	0.9433	No
**PC O-18:1_20:4**	32.83	2.55	11	35.35	2.35	12	29.98	0.2604	0.5152	No

Abbreviations: CON Control; **HD** Huntington's disease; **PC** Phosphatidylcholine; **PD** Percentage Difference; **SEM** Standard Error of Mean.

Table S5 PC Ester Linked Fatty Acids in Control and HD Caudate.

	CON			HD						
	Mean	SEM	N	Mean	SEM	N	PD (%)	p	q	Discovery?
**16:0**	15723.86	1000.78	13	14494.19	863.19	12	24.08	0.3619	0.6292	No
**16:1**	696.94	42.36	13	742.48	56.03	12	28.87	0.5239	0.7208	No
**18:0**	4622.12	370.32	13	3790.68	313.27	12	15.57	0.1002	0.5797	No
**18:1**	12293.71	873.33	13	11463.27	746.60	12	21.19	0.4772	0.7029	No
**18:2**	266.59	21.94	13	272.61	38.73	12	14.22	0.6495	0.7899	No
**18:3**	19.13	1.58	12	24.23	2.84	10	85.11	0.5489	0.7208	No
**20:3**	231.75	17.70	13	214.23	17.50	12	26.32	0.7283	0.8399	No
**20:4**	1753.72	179.36	13	1525.15	128.75	12	35.59	0.3121	0.5911	No
**20:5**	132.62	14.21	13	92.84	14.27	12	-14.34	0.0080	0.1139	No
**22:4**	253.23	26.43	13	206.11	21.02	12	16.72	0.1766	0.5797	No
**22:5**	125.57	11.26	13	107.04	11.04	12	2.66	0.2520	0.5911	No
**22:6**	857.76	100.83	13	692.24	68.12	12	17.67	0.1883	0.5874	No

Abbreviations: CON Control; **HD** Huntington's disease; **PC** Phosphatidylcholine; **PD** Percentage Difference; **SEM** Standard Error of Mean.

Table S6 PC Ether Linked Fatty Acids in Control and HD Caudate.

	CON			HD						
	Mean	SEM	N	Mean	SEM	N	PD (%)	p	q	Discovery?
**O-16:0**	119.98	6.15	13	117.59	9.29	12	28.57	0.8325	0.9387	No
**O-16:1**	42.01	4.71	13	51.59	5.12	12	51.83	0.0523	0.3118	No
**O-18:0**	37.53	2.16	13	37.04	3.13	11	28.97	0.4596	0.7012	No
**O-18:1**	346.26	68.48	13	300.54	37.27	12	0.73	0.5743	0.7440	No

Abbreviations: CON Control; **HD** Huntington's disease; **PC** Phosphatidylcholine; **PD** Percentage Difference; **SEM** Standard Error of Mean.

Table S7 Ester PE Species in Control and HD Caudate.

	CON			HD						
	**Mean**	**SEM**	**N**	**Mean**	**SEM**	**N**	**PD (%)**	**p**	**q**	**Discovery?**
**PE 16:0_18:1**	306.55	19.69	13	379.43	16.77	12	20.30	0.0098	0.1739	No
**PE 16:1_18:0**	12.57	1.45	13	11.13	1.43	12	94.86	0.4857	0.7048	No
**PE 16:0_18:2**	6.31	1.51	13	9.24	1.42	12	-42.48	0.0976	0.3481	No
**PE 16:1_18:1**	31.62	4.41	13	48.82	5.65	12	70.71	0.0096	0.1139	No
**PE 18:0_18:0**	4.90	0.88	11	5.37	0.70	10	-79.74	0.6772	0.8350	No
**PE 18:0_18:1**	368.82	36.08	13	312.89	25.97	12	16.44	0.2219	0.5911	No
**PE 18:0_18:2**	25.68	5.43	13	15.61	3.03	12	-59.05	0.1222	0.5797	No
**PE 18:1_18:1**	329.89	49.67	13	371.77	44.59	12	-12.56	0.2701	0.5152	No
**PE 18:1_18:2**	31.20	3.54	13	48.06	3.37	12	6.99	0.0022	0.0772	No
**PE 16:0_20:4**	126.20	12.31	13	153.96	11.48	12	32.81	0.1095	0.3481	No
**PE 16:0_20:5**	1.51	0.46	12	2.19	0.45	12	-42.62	0.1782	0.3933	No
**PE 16:1_20:4**	12.22	1.05	12	21.44	2.66	12	16.78	0.0029	0.0579	No
**PE 18:0_20:3**	110.56	9.35	13	109.41	12.46	12	37.24	0.9420	0.9792	No
**PE 16:0_22:4**	95.99	9.92	13	80.60	8.17	12	-5.54	0.2436	0.5911	No
**PE 18:0_20:4**	1383.65	163.22	13	1207.28	108.31	12	33.13	0.3784	0.6369	No
**PE 18:1_20:3**	31.35	4.35	13	27.22	4.21	11	-45.64	0.7330	0.8399	No
**PE 16:0_22:5**	99.13	6.03	13	119.34	7.32	12	-0.04	0.0444	0.4040	No
**PE 18:0_20:5**	2.86	0.61	11	3.72	0.99	11	96.65	0.4657	0.7012	No
**PE 18:1_20:4**	219.13	14.51	13	306.42	17.72	12	-9.87	0.0010	0.0642	No
**PE 16:0_22:6**	385.12	52.75	13	370.93	36.33	12	43.67	0.8268	0.9387	No
**PE 16:1_22:5**	6.25	1.34	13	11.13	1.66	11	1410.10	0.0331	0.3506	No
**PE 18:1_20:5**	22.56	5.13	13	17.11	4.98	12	-82.76	0.3475	0.6090	No
**PE 18:2_20:4**	9.06	1.37	13	13.21	2.54	12	307.57	0.1689	0.5797	No
**PE 18:0_22:4**	890.34	119.68	13	737.99	69.37	12	27.11	0.2845	0.5911	No
**PE 18:0_22:5**	230.58	30.83	13	226.30	39.36	12	13.50	0.7689	0.8485	No
**PE 18:1_22:4**	110.01	7.93	13	82.33	10.34	12	11.02	0.0457	0.4040	No
**PE 18:0_22:6**	2870.82	390.78	13	2068.99	223.15	12	3.82	0.0908	0.5797	No
**PE 18:1_22:5**	42.51	8.00	13	53.92	6.80	12	63.53	0.2884	0.5911	No
**PE 18:1_22:6**	213.14	24.92	13	193.58	14.69	12	5.11	0.5068	0.7167	No
**PE 18:2_22:6**	14.33	1.36	10	18.46	1.22	6	-34.20	0.0727	0.3481	No
**PE 20:3_22:6**	49.24	5.83	12	39.84	7.38	11	-31.17	0.2115	0.4501	No

Abbreviations: CON Control; **HD** Huntington's disease; **PD** Percentage Difference; **PE** Phosphatidylethanolamine; **SEM** Standard Error of Mean.

Table S8 Ether PE Species in Control and HD Caudate.

	CON Mean	SEM	N	HD Mean	SEM	N	PD (%)	p	q	Discovery?
**PE O-18:1_16:0**	141.24	33.06	13	121.86	18.62	11	-47.86	>0.999	>0.999	No
**PE O-16:1_18:1**	166.74	63.59	13	140.76	47.31	12	8.30	0.4696	0.7290	No
**PE O-18:2_16:0**	55.90	11.30	13	47.50	14.47	12	-50.77	0.1519	0.3481	No
**PE O-18:1_18:1**	199.55	70.03	13	157.89	40.67	12	-51.00	0.5382	0.7290	No
**PE O-18:1_18:2**	496.60	200.25	11	353.96	149.89	11	-46.36	0.1164	0.3481	No
**PE O-16:1_20:3**	87.46	10.80	13	147.75	14.08	12	50.71	0.0019	0.0579	No
**PE O-16:1_20:4**	65.79	5.28	11	70.89	3.07	9	7.76	0.4158	0.6582	No
**PE O-18:1_20:3**	395.72	25.48	13	390.21	27.82	12	52.05	0.8852	0.9387	No
**PE O-16:1_22:4**	178.93	27.75	13	146.82	33.16	12	-10.77	0.0457	0.3027	No
**PE O-18:1-_20:4**	328.65	33.26	13	323.02	18.79	12	54.03	0.8846	0.9387	No
**PE O-18:2_20:3**	22.95	6.31	13	20.71	6.46	12	-48.37	0.8517	0.9063	No
**PE O-16:1_22:5**	26.94	3.46	13	31.86	3.14	12	-24.02	0.1519	0.3481	No
**PE O-18:1_20:5**	4.08	0.94	13	5.87	1.25	11	-26.80	0.2767	0.5152	No
**PE O-18:2_20:4**	369.50	34.72	13	437.61	39.93	12	-1.42	0.3760	0.6252	No
**PE O-16:1_22:6**	95.58	12.81	11	101.67	12.09	11	-50.54	0.7329	0.8832	No
**PE O-18:1_22:4**	456.85	51.00	13	417.53	35.57	12	25.66	0.5339	0.7208	No
**PE O-18:0_22:6**	21.42	4.01	13	29.42	4.59	12	-9.18	0.2024	0.5911	No
**PE O-18:1_22:5**	87.39	10.63	13	135.41	11.80	12	53.06	0.0062	0.1548	No
**PE O-18:2_22:4**	403.39	46.92	13	334.29	45.49	12	-10.20	0.1366	0.3481	No
**PE O-18:1_22:6**	377.09	47.81	13	379.35	33.64	12	2.49	0.9695	0.9792	No
**PE O-18:2_22:5**	18.93	4.02	13	21.61	2.33	12	63.87	0.0868	0.3481	No
**PE O-18:2_22:6**	181.35	25.75	13	139.67	19.57	12	3.37	0.0868	0.3481	No

Abbreviations: CON Control; **HD** Huntington's disease; **PD** Percentage Difference; **PE** Phosphatidylethanolamine; **SEM** Standard Error of Mean.

Table S9 PE Ester Linked Fatty Acids in Control and HD Caudate.

	CON			HD						
	Mean	SEM	N	Mean	SEM	N	PD (%)	p	q	Discovery?
**16:0**	1217.83	85.80	13	1274.90	51.32	12	13.00	0.9362	0.9787	No
**16:1**	61.73	5.24	13	91.59	6.11	12	78.05	0.0012	0.0642	No
**18:0**	5903.73	725.07	13	4701.98	432.38	12	14.49	0.1705	0.5797	No
**18:1**	2402.97	286.25	13	2509.69	210.31	12	-0.59	0.5033	0.7290	No
**18:2**	503.48	177.51	13	419.82	139.94	12	-40.57	0.4371	0.7040	No
**20:3**	693.50	41.83	13	729.56	48.84	12	32.18	0.5806	0.7509	No
**20:4**	2503.14	232.09	13	2516.11	143.02	12	27.67	0.9625	0.9792	No
**20:5**	30.46	5.57	13	28.09	5.29	12	-46.47	0.7612	0.9071	No
**22:4**	2135.51	199.35	13	1799.56	137.98	12	13.67	0.1804	0.5797	No
**22:5**	511.73	51.23	13	598.64	50.47	12	23.96	0.2391	0.5911	No
**22:6**	4186.30	529.15	13	3320.90	317.86	12	4.90	0.1767	0.5797	No

Abbreviations: CON Control; **HD** Huntington's disease; **PD** Percentage Difference; **PE** Phosphatidylethanolamine; **SEM** Standard Error of Mean.

Table S10 PE Ether Linked Fatty Acids in Control and HD Caudate.

	CON			HD						
	Mean	SEM	N	Mean	SEM	N	PD (%)	p	q	Discovery?
**O-16:1**	596.62	104.59	13	613.55	85.60	12	3.58	0.3777	0.6252	No
**O-18:0**	21.42	4.01	13	29.42	4.59	12	-9.18	0.2024	0.5911	No
**O-18:1**	2410.78	319.58	13	2244.97	217.94	12	7.92	0.5382	0.7290	No
**O-18:2**	1052.02	120.30	13	1001.39	120.30	12	-6.85	0.7689	0.8485	No

Abbreviations: CON Control; **HD** Huntington's disease; **PD** Percentage Difference; **PE** Phosphatidylethanolamine; **SEM** Standard Error of Mean.

Table S11 PS Species in Control and HD Caudate.

	CON			HD						
	**Mean**	**SEM**	**N**	**Mean**	**SEM**	**N**	**PD (%)**	**p**	**q**	**Discovery?**
**PS 18:0_18:1**	1665.83	149.96	12	1242.37	95.40	12	-29.73	0.0280	0.3506	No
**PS 18:1_18:1**	285.30	23.06	12	291.33	23.71	12	-40.04	0.8571	0.9387	No
**PS 18:0_20:3**	91.94	8.45	12	113.00	9.71	9	33.61	0.1198	0.5797	No
**PS 18:0_20:4**	193.39	13.10	12	205.19	13.77	10	-36.43	0.5416	0.7208	No
**PS 18:0_22:4**	473.13	48.93	12	462.35	48.10	12	25.31	0.8766	0.9387	No
**PS 18:0_22:5**	204.95	22.93	12	290.76	48.45	10	103.45	0.1402	0.3481	No
**PS 18:0_22:6**	2192.35	261.71	12	2008.44	244.04	12	23.05	0.6124	0.7732	No

Abbreviations: CON Control; **HD** Huntington's disease; **PD** Percentage Difference; **PS** Phosphatidylserine; **SEM** Standard Error of Mean.

Table S12 PS Derived Fatty Acids in Control and HD Caudate.

	CON			HD						
	**Mean**	**SEM**	**N**	**Mean**	**SEM**	**N**	**PD (%)**	**p**	**q**	**Discovery?**
**18:0**	4821.59	332.10	12	4211.21	403.14	12	3.63	0.2555	0.5911	No
**18:1**	2236.43	193.53	12	1825.03	134.72	12	-32.52	0.0967	0.5797	No
**20:3**	91.94	8.45	12	113.00	9.71	9	33.61	0.1198	0.5797	No
**20:4**	193.39	13.10	12	205.19	13.77	10	-36.43	0.5416	0.7208	No
**22:4**	473.13	48.93	12	462.35	48.10	12	25.31	0.8766	0.9387	No
**22:5**	204.95	22.93	12	290.76	48.45	10	103.45	0.1402	0.3481	No
**22:6**	2192.35	261.71	12	2008.44	244.04	12	23.05	0.6124	0.7732	No

Abbreviations: CON Control; **HD** Huntington's disease; **PD** Percentage Difference; **PS** Phosphatidylserine; **SEM** Standard Error of Mean.

Table S13 Total Ester Linked Phospholipid Derived Fatty Acids in Control and HD Caudate.

	CON Mean	SEM	N	HD Mean	SEM	N	PD (%)	p	q	Discovery?
**16:0**	16.81	1.15	12	15.55	0.96	11	-7.48	0.4119	0.6582	No
**16:1**	0.73	0.04	12	0.79	0.05	11	8.99	0.2826	0.5911	No
**18:0**	15.55	1.42	12	12.99	1.03	11	-16.44	0.1611	0.5797	No
**18:1**	16.10	0.85	12	15.17	0.65	11	-5.81	0.3934	0.6519	No
**18:2**	0.60	0.05	12	0.55	0.05	11	-8.42	0.4918	0.7048	No
**18:3**	0.02	0.00	12	0.02	0.00	9	29.03	0.1400	0.5797	No
**20:3**	0.99	0.06	12	1.01	0.07	11	1.62	0.8605	0.9387	No
**20:4**	4.59	0.41	12	4.28	0.30	11	-6.76	0.5505	0.7208	No
**20:5**	0.15	0.01	12	0.11	0.01	11	-27.89	0.0073	0.1548	No
**22:4**	2.83	0.28	12	2.43	0.18	11	-13.93	0.2567	0.5911	No
**22:5**	0.85	0.08	12	0.98	0.11	11	15.73	0.3542	0.6261	No
**22:6**	7.45	0.89	12	6.31	0.59	11	-15.30	0.2993	0.5911	No
**Total Saturated**	33.01	2.67	12	28.58	2.39	9	-13.44	0.2502	0.5911	No
**Total Monounsaturated**	17.78	1.34	12	16.02	0.86	9	-9.87	0.4865	0.7290	No
**Total Polyunsaturated**	17.27	1.69	12	15.53	1.43	9	-10.06	0.4000	0.6526	No
**Total Phospholipid**	68.06	5.27	12	60.13	4.57	9	-11.65	0.3103	0.5911	No

Abbreviations: CON Control; **HD** Huntington's disease; **PD** Percentage Difference; **SEM** Standard Error of Mean.

Table S14 Phospholipid Class Totals in Control and HD Caudate.

	CON Mean	SEM	N	HD Mean	SEM	N	PD (%)	p	q	Discovery?
**Total Ester PC**	18214.87	1187.31	13	16558.68	1019.03	12	22.31	0.3009	0.5911	No
**Total Ether PC**	545.78	75.12	13	503.68	50.50	12	13.56	0.6114	0.7752	No
**Total Ester PE**	8034.76	837.44	13	7050.75	476.51	12	13.64	0.3200	0.5954	No
**Total Ether PE**	4080.83	535.07	13	3889.34	416.19	12	2.59	0.5743	0.7440	No
**Total PS (Ester)**	5106.90	339.73	12	4502.53	407.56	12	0.90	0.2673	0.5911	No

Abbreviations: CON Control; **HD** Huntington's disease; **PC** Phosphatidylcholine; **PD** Percentage Difference; **PE** Phosphatidylethanolamine; **PS** Phosphatidylserine; **SEM** Standard Error of Mean.

Table S15 LPC and LPE species in control and HD caudate.

	CON Mean	SEM	N	HD Mean	SEM	N	PD (%)	p	q	Discovery?
**LPC 16:0**	50.39	2.18	13	40.16	3.86	13	-20.29	0.03	0.16	No
**LPC 16:1**	1.72	0.24	9	1.89	0.43	7	9.58	0.76	0.84	No
**LPC 18:0**	23.60	1.55	13	18.29	1.23	12	-22.51	0.01	0.07	No
**LPC 18:1**	42.64	2.18	13	35.61	3.20	13	-16.48	0.08	0.21	No
**LPC 18:2**	2.58	0.68	6	2.17	0.51	7	-16.06	0.84	0.84	No
**LPC 20:4**	8.10	1.10	13	5.74	0.58	13	-29.14	0.07	0.21	No
**LPC 22:4**	1.77	0.34	10	1.77	0.25	8	0.17	1.00	>0.9999	No
**LPC 22:6**	4.62	0.64	13	10.91	5.55	12	136.01	0.61	0.84	No
**Total LPC**	133.10	6.65	13	111.74	6.44	13	-16.05	0.03	0.16	No
**LPE 16:0**	23.48	1.19	13	26.48	2.60	13	12.77	0.31	0.42	No
**LPE 16:1**	15.63	6.01	6	10.44	2.79	7	-33.20	0.73	0.84	No
**LPE 18:0**	105.04	13.14	13	87.92	9.34	12	-16.30	0.30	0.42	No
**LPE 18:1**	92.54	24.50	13	78.31	18.60	13	-15.38	0.65	0.84	No
**LPE 22:5**	11.52	1.80	10	16.10	3.35	8	39.80	0.25	0.42	No
**LPE 22:6**	79.99	12.38	13	70.10	10.26	13	-12.36	0.54	0.61	No
**Total LPE**	317.12	30.20	13	271.58	34.41	13	-14.36	0.33	0.42	No

Abbreviations: CON Control; **HD** Huntington’s disease; **LPC** Lysophosphatidylcholine; **LPE** Lysophophatidylethanolamine; **PD** Percentage Difference; **SEM** Standard Error of Mean.

3.3.2 Putamen

Table S16 Ester PC Species in Control and HD Putamen.

	CON Mean	SEM	N	HD Mean	SEM	N	PD (%)	p	q	Discovery?
**PC 16:0_16:0**	2703.22	161.11	12	1889.32	113.41	13	-30.11	0.0005	0.0026	Yes
**PC 16:0_16:1**	544.80	32.85	12	692.64	48.23	13	27.14	0.0678	0.1238	No
**PC 16:0_18:0**	14227.34	1680.32	12	6885.94	1199.94	13	-51.60	0.0020	0.0061	Yes
**PC 16:0_18:1**	9926.26	447.70	12	9466.07	422.48	13	-4.64	0.4371	0.5320	No
**PC 16:1_18:0**	106.87	2.46	12	112.84	6.17	13	5.58	0.3829	0.3625	No
**PC 16:0_18:2**	188.17	11.14	12	193.32	15.39	13	2.74	1.0000	0.9890	No
**PC 16:1_18:1**	200.57	14.58	12	279.18	19.17	13	39.19	0.0045	0.0214	No
**PC 18:0_18:1**	3231.13	175.24	12	2803.66	158.13	13	-13.23	0.0976	0.1717	No
**PC 18:0_18:2**	99.30	5.41	12	83.16	7.36	13	-16.25	0.0915	0.1190	No
**PC 18:1_18:1**	970.00	99.33	12	931.68	69.35	13	-3.95	0.8938	0.9224	No
**PC 16:0_20:3**	127.71	9.73	12	98.92	7.57	13	-22.54	0.0294	0.0468	No
**PC 18:1_18:2**	62.72	4.43	12	72.40	6.11	12	15.43	0.2142	0.2327	No
**PC 16:0_20:4**	862.76	58.12	12	596.33	43.79	13	-30.88	0.0015	0.0053	Yes
**PC 18:0_20:3**	89.36	5.62	12	87.24	4.99	13	-2.37	0.7807	0.6264	No
**PC 16:0_22:4**	145.62	8.42	12	92.86	10.35	13	-36.23	0.0007	0.0028	Yes
**PC 18:0_20:4**	1008.21	80.11	12	567.62	69.09	13	-43.70	0.0004	0.0023	Yes
**PC 18:1_20:3**	51.18	4.60	12	62.93	6.61	11	22.96	0.1618	0.1828	No
**PC 18:0_20:5**	126.73	11.12	12	123.15	8.28	13	-2.83	0.7985	0.6289	No
**PC 18:1_20:4**	204.89	23.07	12	116.71	12.25	13	-43.04	0.0036	0.0097	Yes
**PC 16:0_22:6**	547.41	43.62	12	336.08	27.07	13	-38.60	0.0006	0.0028	Yes
**PC 18:0_22:4**	185.70	11.40	12	134.51	9.94	8	-27.57	0.0033	0.0092	Yes
**PC 18:0_22:6**	437.98	31.17	12	260.26	16.94	13	-40.58	0.0001	0.0010	Yes
**PC 18:1_22:6**	200.69	11.18	12	121.04	6.95	13	-39.69	0.0000	0.0006	Yes

Abbreviations: CON Control; **HD** Huntington's disease; **PC** Phosphatidylcholine; **PD** Percentage Difference; **SEM** Standard Error of Mean.

Table S17 Ether PC Species in Control and HD Putamen.

	CON Mean	SEM	N	HD Mean	SEM	N	PD (%)	p	q	Discovery?
**PC O-16:0_16:0**	105.91	5.32	12	99.48	5.57	13	-6.07	0.4126	0.3809	No
**PC O-16:1_16:0**	45.40	7.04	12	58.76	5.80	13	29.41	0.0160	0.0487	No
**PC O-18:1_16:0**	457.58	108.74	12	474.31	52.17	13	3.65	0.1519	0.2575	No
**PC O-16:0_20:4**	39.64	3.02	12	38.13	1.47	13	-3.82	0.8938	0.9224	No
**PC O-16:1_20:4**	13.97	0.90	10	13.42	0.79	12	-3.91	0.6535	0.5611	No
**PC O-18:0_20:4**	51.44	2.62	12	39.30	2.56	12	-23.60	0.0031	0.0090	Yes
**PC O-18:1_20:4**	46.73	2.33	12	44.16	1.94	12	-5.49	0.4696	0.5573	No

Abbreviations: CON Control; **HD** Huntington's disease; **PC** Phosphatidylcholine; **PD** Percentage Difference; **SEM** Standard Error of Mean.

Table S18 PC Ester Linked Fatty Acids in Control and HD Putamen.

	CON Mean	SEM	N	HD Mean	SEM	N	PD (%)	p	q	Discovery?
**16:0**	32585.41	1581.80	12	22773.35	1368.83	13	-30.11	0.0001	0.0010	Yes
**16:1**	852.24	47.29	12	1084.65	68.44	13	27.27	0.0345	0.0780	No
**18:0**	19512.61	1609.29	12	11006.65	1295.38	13	-43.59	0.0005	0.0025	Yes
**18:1**	15817.45	795.87	12	14770.10	697.79	13	-6.62	0.2945	0.3995	No
**18:2**	350.19	17.53	12	343.31	27.72	13	-1.96	0.4371	0.5298	No
**20:3**	268.25	10.96	12	239.41	15.82	13	-10.75	0.1489	0.1704	No
**20:4**	2225.32	157.48	12	1408.22	125.60	13	-36.72	0.0005	0.0026	Yes
**20:5**	126.73	11.12	12	123.15	8.28	13	-2.83	0.7985	0.6289	No
**22:4**	331.32	19.23	12	175.63	26.06	13	-46.99	0.0001	0.0010	Yes
**22:6**	1186.08	84.71	12	717.39	45.91	13	-39.52	0.0001	0.0012	Yes

Abbreviations: CON Control; **HD** Huntington's disease; **PC** Phosphatidylcholine; **PD** Percentage Difference; **SEM** Standard Error of Mean.

Table S19 PC Ether Linked Fatty Acids in Control and HD Putamen.

	CON			HD						
	**Mean**	**SEM**	**N**	**Mean**	**SEM**	**N**	**PD (%)**	**p**	**q**	**Discovery?**
**O-16:0**	145.56	6.42	12	137.61	6.25	13	-5.46	0.3843	0.3625	No
**O-16:1**	57.04	7.82	12	71.15	5.70	13	24.73	0.0257	0.0609	No
**O-18:0**	51.44	2.62	12	39.30	2.56	12	-23.60	0.0045	0.0214	No
**O-18:1**	504.31	110.65	12	515.07	51.78	13	2.13	0.1683	0.2756	No

Abbreviations: CON Control; **HD** Huntington's disease; **PC** Phosphatidylcholine; **PD** Percentage Difference; **SEM** Standard Error of Mean.

Table S20 Ester PE Species in Control and HD Putamen.

	CON Mean	SEM	N	HD Mean	SEM	N	PD (%)	p	q	Discovery?
**PE 16:0_18:1**	413.93	17.53	12	589.41	48.35	13	42.39	0.0038	0.0099	Yes
**PE 16:1_18:0**	23.97	1.05	12	30.07	1.59	13	25.42	0.0044	0.0105	No
**PE 16:0_18:2**	8.48	1.15	12	11.80	1.16	13	39.23	0.0531	0.0735	No
**PE 16:1_18:1**	44.67	2.83	12	92.31	9.74	13	106.66	0.0003	0.0023	Yes
**PE 18:0_18:1**	624.29	20.76	12	544.20	45.61	13	-12.83	0.1287	0.1535	No
**PE 18:0_18:2**	36.36	3.13	12	29.16	3.96	13	-19.80	0.1678	0.1871	No
**PE 18:1_18:1**	471.94	35.97	12	764.44	95.93	13	61.98	0.0119	0.0246	No
**PE 16:0_20:3**	25.56	2.19	12	28.55	2.13	13	11.71	0.3368	0.3402	No
**PE 18:1_18:2**	49.64	4.60	12	71.94	4.14	13	44.92	0.0015	0.0053	Yes
**PE 16:0_20:4**	179.27	12.33	12	193.68	16.09	13	8.04	0.4847	0.4380	No
**PE 16:0_20:5**	1.46	0.19	10	2.14	0.27	11	45.99	0.0548	0.0746	No
**PE 16:1_20:4**	15.85	1.33	12	25.48	3.10	13	60.79	0.0114	0.0245	No
**PE 18:0_20:3**	160.19	12.77	12	160.59	11.74	13	0.25	0.9787	0.9885	No
**PE 16:0_22:4**	147.22	8.50	12	115.06	9.05	13	-21.84	0.0164	0.0287	No
**PE 18:0_20:4**	2056.09	164.69	12	1223.67	172.77	13	-40.49	0.0020	0.0061	Yes
**PE 18:1_20:3**	36.51	2.05	12	60.07	6.55	13	64.53	0.0039	0.0099	Yes
**PE 16:0_22:5**	124.54	6.03	12	141.17	7.02	13	13.35	0.0398	0.0859	No
**PE 18:0_20:5**	4.65	0.74	12	5.11	1.33	8	9.99	0.7660	0.6204	No
**PE 18:1_20:4**	327.84	14.58	12	392.40	32.20	13	19.69	0.0857	0.1132	No
**PE 16:0_22:6**	589.74	43.35	12	384.62	38.80	13	-34.78	0.0019	0.0061	Yes
**PE 16:1_22:5**	14.80	1.79	12	21.79	1.48	13	47.27	0.0065	0.0146	No
**PE 18:1_20:5**	25.23	1.94	12	34.04	4.87	13	34.91	0.1130	0.1406	No
**PE 18:2_20:4**	22.03	1.97	12	24.29	1.69	13	10.26	0.3923	0.3661	No
**PE 16:1_22:6**	17.51	1.40	12	17.14	1.59	13	-2.11	0.8630	0.6615	No
**PE 18:0_22:4**	1292.16	97.34	12	747.32	99.09	13	-42.17	0.0010	0.0119	No
**PE 18:0_22:5**	339.55	32.34	12	219.75	41.24	13	-35.28	0.0457	0.0943	No
**PE 18:1_22:4**	183.36	10.57	12	156.97	13.35	13	-14.40	0.1352	0.1590	No
**PE 18:0_22:6**	4506.41	308.06	12	2255.76	259.57	13	-49.94	0.0000	0.0006	Yes
**PE 18:1_22:5**	69.92	5.24	12	74.68	5.30	13	6.81	0.4371	0.5320	No
**PE 18:1_22:6**	357.03	18.49	12	264.25	21.07	13	-25.99	0.0031	0.0090	Yes
**PE 18:2_22:6**	25.65	1.25	11	27.48	4.49	9	7.16	0.7021	0.5852	No

Table S20 Continued… Ester PE Species in Control and HD Putamen.

	CON Mean	SEM	N	HD Mean	SEM	N	PD (%)	p	q	Discovery?
**PE 18:2_22:6**	25.65	1.25	11	27.48	4.49	9	7.16	0.7021	0.5852	No
**PE 20:4_22:6**	22.65	1.71	12	16.96	1.36	13	-25.10	0.0164	0.0287	No
**PE 20:3_22:6**	88.28	10.48	12	56.17	8.11	13	-36.37	0.0244	0.0395	No

Abbreviations: CON Control; **HD** Huntington's disease; **PD** Percentage Difference; **PE** Phosphatidylethanolamine; **SEM** Standard Error of Mean.

Table S21 Ether PE Species in Control and HD Putamen.

	CON Mean	SEM	N	HD Mean	SEM	N	PD (%)	p	q	Discovery?
**PE O-18:1_16:0**	164.58	23.13	12	222.57	28.52	13	35.24	0.1283	0.1535	No
**PE O-16:1_18:1**	188.15	30.48	12	385.50	71.39	13	104.89	0.0216	0.0357	No
**PE O-18:2_16:0**	79.68	8.07	12	136.98	18.26	13	71.90	0.0109	0.0240	No
**PE O-18:1_18:1**	249.33	44.17	12	405.22	61.63	13	62.52	0.0597	0.1180	No
**PE O-18:1_18:2**	550.35	97.42	12	1000.52	183.90	13	81.80	0.0441	0.0626	No
**PE O-16:1_20:3**	106.89	10.80	12	143.97	13.62	13	34.69	0.0441	0.0626	No
**PE O-16:1_20:4**	64.24	3.26	12	106.85	14.37	13	66.34	0.0124	0.0248	No
**PE O-18:0_20:4**	51.51	12.18	7	45.38	16.27	9	-11.90	0.5360	0.6058	No
**PE O-18:1_20:3**	514.72	28.08	12	506.62	44.63	13	-1.57	0.8795	0.6682	No
**PE O-16:1_22:4**	227.77	20.00	12	245.51	24.75	13	7.79	0.7283	0.8041	No
**PE O-18:1_20:4**	405.83	24.74	12	341.57	28.62	13	-15.83	0.1030	0.1315	No
**PE O-18:2_20:3**	25.18	3.83	12	65.64	10.66	13	160.66	0.0012	0.0119	No
**PE O-16:1_22:5**	33.29	2.14	12	37.66	3.60	13	13.15	0.3084	0.3186	No
**PE O-18:1_20:5**	5.88	0.97	12	5.08	1.06	13	-13.60	0.5033	0.5827	No
**PE O-18:2_20:4**	539.62	23.60	12	622.30	49.18	13	15.32	0.1478	0.1704	No
**PE O-16:1_22:6**	141.03	12.78	12	134.45	17.65	13	-4.67	0.7656	0.6204	No
**PE O-18:1_22:4**	637.53	36.98	12	536.16	50.58	13	-15.90	0.0257	0.0609	No
**PE O-18:0_22:6**	28.78	5.45	12	17.28	3.99	13	-39.97	0.0678	0.1238	No
**PE O-18:1_22:5**	119.82	16.26	12	93.67	13.25	13	-21.82	0.2051	0.3140	No
**PE O-18:2_22:4**	656.25	35.78	12	586.31	52.27	13	-10.66	0.2821	0.2990	No
**PE O-18:1_22:6**	608.66	27.82	12	396.68	45.15	13	-34.83	0.0007	0.0030	Yes
**PE O-18:2_22:5**	25.26	2.13	12	42.73	5.95	13	69.18	0.0045	0.0214	No
**PE O-18:2_22:6**	275.52	19.57	12	299.16	29.69	13	8.58	0.5136	0.4499	No

Abbreviations: CON Control; **HD** Huntington’s disease; **PD** Percentage Difference; **PE** Phosphatidylethanolamine; **SEM** Standard Error of Mean.

Table S22 PE Ester Linked Fatty Acids in Control and HD Putamen.

	CON			HD						
	**Mean**	**SEM**	**N**	**Mean**	**SEM**	**N**	**PD (%)**	**p**	**q**	**Discovery?**
**16:0**	1734.22	72.82	12	1825.64	110.01	13	5.27	0.4961	0.4437	No
**16:1**	116.79	5.07	12	186.79	14.56	13	59.93	0.0004	0.0023	Yes
**18:0**	9043.68	590.17	12	5213.67	588.96	13	-42.35	0.0002	0.0092	Yes
**18:1**	3513.77	164.69	12	4599.86	426.54	13	30.91	0.0308	0.0473	No
**18:2**	690.37	97.83	12	1156.74	184.73	13	67.55	0.0385	0.0581	No
**20:3**	957.33	36.17	12	1021.62	71.95	13	6.72	0.4353	0.3976	No
**20:4**	3640.81	201.87	12	2961.65	242.22	13	-18.65	0.0422	0.0624	No
**20:5**	36.98	1.76	12	44.08	5.44	13	19.18	0.2341	0.2512	No
**22:4**	2960.94	129.75	12	2230.36	187.65	13	-24.67	0.0043	0.0105	No
**22:5**	727.18	57.56	12	631.46	52.18	13	-13.16	0.2471	0.3665	No
**22:6**	6659.11	389.69	12	3861.50	381.19	13	-42.01	0.0000	0.0006	Yes

Abbreviations: CON Control; **HD** Huntington’s disease; **PD** Percentage Difference; **PE** Phosphatidylethanolamine; **SEM** Standard Error of Mean.

Table S23 PE Ether Linked Fatty Acids in Control and HD Putamen.

	CON			HD						
	**Mean**	**SEM**	**N**	**Mean**	**SEM**	**N**	**PD (%)**	**p**	**q**	**Discovery?**
**O-16:1**	761.36	64.15	12	1053.94	119.44	13	38.43	0.0445	0.0626	No
**O-18:0**	58.83	10.66	12	48.70	13.83	13	-17.23	0.5675	0.4921	No
**O-18:1**	3256.70	191.37	12	3508.08	317.22	13	7.72	0.5054	0.4473	No
**O-18:2**	1601.51	78.91	12	1753.12	144.83	13	9.47	0.3699	0.3609	No

Abbreviations: CON Control; **HD** Huntington’s disease; **PD** Percentage Difference; **PE** Phosphatidylethanolamine; **SEM** Standard Error of Mean.

Table S24 PS Species in Control and HD Putamen.

	CON Mean	SEM	N	HD Mean	SEM	N	PD (%)	p	q	Discovery?
**PS 16:0_18:1**	145.70	15.50	12	244.10	33.35	13	67.53	0.0160	0.0287	No
**PS 18:0_18:1**	2874.12	378.62	12	3977.20	528.27	13	38.38	0.1042	0.1315	No
**PS 18:0_18:2**	25.09	3.47	12	41.58	6.79	13	65.71	0.0257	0.0609	No
**PS 18:1_18:1**	435.16	49.77	12	731.38	105.88	13	68.07	0.0215	0.0357	No
**PS 16:0_20:3**	15.34	2.35	9	19.83	2.42	12	29.26	0.1991	0.2191	No
**PS 16:0_20:4**	17.57	3.34	8	22.20	2.70	9	26.33	0.2997	0.3138	No
**PS 18:0_20:3**	123.46	6.48	12	179.49	19.17	13	45.38	0.0146	0.0277	No
**PS 18:0_20:4**	280.14	20.64	12	271.40	18.64	13	-3.12	0.7563	0.6204	No
**PS 18:1_20:4**	32.37	3.39	12	44.63	2.93	13	37.89	0.0120	0.0246	No
**PS 16:0_22:6**	25.36	3.10	11	29.19	3.94	12	15.12	0.7859	0.8479	No
**PS 18:0_22:4**	651.54	24.14	12	475.79	40.61	13	-26.97	0.0014	0.0053	Yes
**PS 18:0_22:5**	273.08	18.63	12	238.03	33.23	13	-12.84	0.3692	0.3609	No
**PS 18:0_22:6**	2758.15	170.88	12	1671.41	192.37	13	-39.40	0.0003	0.0023	Yes

Abbreviations: CON Control; **HD** Huntington's disease; **PD** Percentage Difference; **PS** Phosphatidylserine; **SEM** Standard Error of Mean.

Table S25 PS Fatty Acids in Control and HD Putamen.

	CON			HD						
	**Mean**	**SEM**	**N**	**Mean**	**SEM**	**N**	**PD (%)**	**p**	**q**	**Discovery?**
**16:0**	192.17	21.03	12	304.71	38.17	13	58.57	0.0185	0.0318	No
**18:0**	6985.58	332.24	12	6854.89	526.85	13	-1.87	0.8359	0.6465	No
**18:1**	3922.51	474.48	12	5728.69	754.99	13	46.05	0.0564	0.0756	No
**18:2**	25.09	3.47	12	41.58	6.79	13	65.71	0.0257	0.0609	No
**20:3**	134.96	7.66	12	197.79	21.01	13	46.55	0.0131	0.0256	No
**20:4**	324.22	23.38	12	331.40	18.81	13	2.21	0.8132	0.6346	No
**22:4**	651.54	24.14	12	475.79	40.61	13	-26.97	0.0014	0.0053	Yes
**22:5**	273.08	18.63	12	238.03	33.23	13	-12.84	0.3692	0.3609	No
**22:6**	2781.40	174.00	12	1698.35	192.26	13	-38.94	0.0004	0.0023	Yes

Abbreviations: CON Control; **HD** Huntington's disease; **PD** Percentage Difference; **PS** Phosphatidylserine; **SEM** Standard Error of Mean.

Table S26 Total Phospholipid Derived Fatty Acids in Control and HD Putamen.

	CON			HD						
	Mean	SEM	N	Mean	SEM	N	PD (%)	p	q	Discovery?
**16:0**	31.86	2.94	13	24.90	1.38	13	-21.83	0.0086	0.0314	No
**16:1**	0.89	0.08	13	1.27	0.08	13	42.14	0.0016	0.0123	No
**18:0**	32.81	2.79	13	23.08	1.92	13	-29.67	0.0061	0.0240	No
**18:1**	21.46	1.21	13	25.10	1.76	13	16.93	0.2869	0.3995	No
**18:2**	0.98	0.10	13	1.54	0.18	13	56.72	0.0148	0.0277	No
**20:3**	1.26	0.10	13	1.46	0.09	13	16.16	0.2035	0.3140	No
**20:4**	5.71	0.52	13	4.70	0.35	13	-17.73	0.1228	0.1506	No
**20:5**	151.12	13.48	13	167.23	12.16	13	10.66	0.3839	0.3625	No
**22:4**	3.64	0.32	13	2.88	0.23	13	-20.84	0.0140	0.0476	No
**22:5**	0.92	0.10	13	0.87	0.08	13	-5.83	0.3897	0.5138	No
**22:6**	9.81	0.96	13	6.28	0.59	13	-36.01	0.0012	0.0119	No
**Total Saturated**	33.79	2.80	13	24.62	1.92	13	-27.15	0.0051	0.0219	No
**Total Monounsaturated**	21.46	1.21	13	25.10	1.76	13	16.93	0.2869	0.3995	No
**Total Polyunsaturated**	21.49	1.91	13	16.36	1.24	13	-23.91	0.0042	0.0214	No
**Total Phospholipid**	76.75	4.82	13	66.07	3.51	13	-13.91	0.0164	0.0487	No

Abbreviations: CON Control; **HD** Huntington's disease; **PD** Percentage Difference; **SEM** Standard Error of Mean.

Table S27 Phospholipid Class Totals in Control and HD Putamen.

	CON			HD						
	Mean	SEM	N	Mean	SEM	N	PD (%)	p	q	Discovery?
**Total Ester PC**	36248.62	1480.35	12	25940.88	1415.30	13	-28.44	0.0000	0.0006	Yes
**Total Ether PC**	758.35	122.87	12	760.10	59.79	13	0.23	0.4059	0.5208	No
**Total Ester PE**	12304.40	670.80	12	8771.73	673.31	13	-28.71	0.0010	0.0119	No
**Total Ether PE**	5678.41	324.75	12	6363.84	573.87	13	12.07	0.3118	0.3186	No
**Total PS (Ester)**	7645.28	378.45	12	7935.62	626.60	13	3.80	0.6959	0.5852	No

Abbreviations: CON Control; **HD** Huntington's disease; **PC** Phosphatidylcholine; **PD** Percentage Difference; **PE** Phosphatidylethanolamine; **PS** Phosphatidylserine; **SEM** Standard Error of Mean.

Table S28 Confirmation Analysis of Origin of 22:6 Reductions in PE Species.

	CON			HD						
**Confirmation Analysis**	**Mean**	**SEM**	**N**	**Mean**	**SEM**	**N**	**PD (%)**	**p**	**q**	**Discovery?**
**PE Ether Species 22:6**	972.92	91.18	13	847.57	71.93	13	-12.88	0.0989	0.0512	No
**PE Ester Species 22:6**	5195.65	551.03	13	3032.95	323.37	13	-41.63	0.0012	0.0013	Yes

Abbreviations: CON Control; **HD** Huntington's disease; **PD** Percentage Difference; **PE** Phosphatidylethanolamine; **SEM** Standard Error of Mean.

Table S29 LPC and LPE species in control and HD putamen.

	CON			HD						
	**Mean**	**SEM**	**N**	**Mean**	**SEM**	**N**	**PD (%)**	**p**	**q**	**Discovery?**
**LPC 16:0**	48.17	2.92	13	36.71	2.13	13	-23.78	0.00	0.00	Yes
**LPC 16:1**	2.22	0.12	13	2.51	0.23	13	13.20	0.27	0.17	No
**LPC 18:0**	24.97	1.44	13	12.81	0.77	13	-48.70	<0.000001	0.00	Yes
**LPC 18:1**	48.08	2.70	13	36.99	2.41	13	-23.06	0.00	0.00	Yes
**LPC 18:2**	1.16	0.17	12	1.48	0.20	12	26.92	0.25	0.17	No
**LPC 20:4**	8.74	0.81	13	5.24	0.28	13	-40.02	0.00	0.00	Yes
**LPC 22:4**	2.79	0.41	13	1.53	0.34	13	-45.38	0.03	0.03	No
**LPC 22:6**	8.75	2.76	13	3.61	0.48	13	-58.70	0.00	0.00	Yes
**Total LPC**	144.80	8.99	13	100.77	5.05	13	-30.41	0.00	0.00	Yes
**LPE 16:0**	34.39	3.55	13	42.98	5.73	13	24.96	0.22	0.17	No
**LPE 16:1**	11.62	2.35	9	15.71	2.87	11	35.16	0.33	0.20	No
**LPE 18:0**	113.75	8.71	13	66.63	7.12	13	-41.43	0.00	0.00	Yes
**LPE 18:1**	223.38	44.65	13	306.95	64.38	13	37.41	0.51	0.26	No
**LPE 22:5**	19.18	3.74	13	14.54	3.36	13	-24.17	0.27	0.20	No
**LPE 22:6**	175.63	25.23	13	96.76	15.34	13	-44.91	0.01	0.02	No
**Total LPE**	574.38	67.27	13	541.14	81.68	13	-5.79	0.76	0.38	No

Abbreviations: CON Control; **HD** Huntington's disease; **LPC** Lysophosphatidylcholine; **LPE** Lysophophatidylethanolamine; **PD** Percentage Difference; **SEM** Standard Error of Mean.

3.3.3 White Matter of the Dorsomedial Prefrontal Cortex

Table S30 Ester PC Species in Control and HD White Cortex.

	CON Mean	SEM	N	HD Mean	SEM	N	PD (%)	p	q	Discovery?
**PC 16:0_16:0**	1720.19	85.71	11	1449.18	138.51	11	-15.8	0.1148	0.1328	No
**PC 16:0_16:1**	745.98	36.63	11	587.83	36.41	11	-21.2	0.0062	0.0169	No
**PC 16:0_18:0**	85.30	16.11	10	128.34	35.19	8	50.5	0.2923	0.2952	No
**PC 16:0_18:1**	12393.37	674.33	11	9126.74	581.03	11	-26.4	0.0002	0.0005	Yes
**PC 16:1_18:0**	164.00	10.19	11	127.07	9.39	11	-22.5	0.0150	0.0341	No
**PC 16:0_18:2**	127.15	6.77	11	116.50	12.27	11	-8.4	0.4586	0.4111	No
**PC 16:1_18:1**	371.10	16.53	11	258.24	18.87	11	-30.4	0.0002	0.0030	Yes
**PC 18:0_18:1**	5440.54	361.15	11	3551.59	272.27	11	-34.7	0.0005	0.0033	Yes
**PC 18:0_18:2**	115.22	7.38	11	82.95	10.44	11	-28.0	0.0213	0.0424	No
**PC 18:1_18:1**	2262.48	104.79	11	1275.31	123.82	11	-43.6	0.0000	0.0005	Yes
**PC 16:0_20:3**	67.69	4.98	11	61.37	5.98	11	-9.3	0.3653	0.2788	No
**PC 18:1_18:2**	98.13	4.60	11	72.77	9.63	11	-25.9	0.0066	0.0108	No
**PC 16:0_20:4**	407.44	19.00	11	330.20	35.68	11	-19.0	0.0750	0.0981	No
**PC 16:1_20:4**	28.06	2.94	11	19.69	3.48	8	-29.8	0.0860	0.1056	No
**PC 18:0_20:3**	42.00	3.25	8	36.78	4.34	8	-12.4	0.1949	0.1698	No
**PC 16:0_22:4**	78.84	5.17	11	52.44	7.17	11	-33.5	0.0079	0.0209	No
**PC 18:0_20:4**	311.88	20.36	11	276.42	36.95	11	-11.4	0.4134	0.3792	No
**PC 18:1_20:3**	144.50	7.36	11	98.23	11.15	11	-32.0	0.0029	0.0083	Yes
**PC 16:0_22:5**	7.32	0.88	11	7.61	1.69	11	4.0	0.8812	0.6760	No
**PC 18:0_20:5**	188.50	11.31	11	127.17	11.29	11	-32.5	0.0010	0.0043	Yes
**PC 18:1_20:4**	53.84	3.70	11	37.49	7.34	11	-30.4	0.0655	0.0886	No
**PC 16:0_22:6**	88.71	8.74	11	86.41	21.06	11	-2.6	0.2169	0.1790	No
**PC 18:1_20:5**	112.82	6.70	11	67.79	5.83	11	-39.9	0.0001	0.0010	Yes
**PC 16:1_22:6**	31.27	1.49	11	25.19	2.78	8	-19.4	0.0804	0.1032	No
**PC 18:0_22:4**	94.34	6.28	11	69.53	6.30	9	-26.3	0.0123	0.0306	No
**PC 18:0_22:5**	71.03	3.07	6	53.17	4.24	8	-25.2	0.0047	0.0082	Yes
**PC 18:0_22:6**	91.76	5.94	11	69.11	11.24	11	-24.7	0.0948	0.1129	No
**PC 18:1_22:5**	44.73	3.49	11	27.52	2.39	11	-38.5	0.0007	0.0033	Yes
**PC 18:1_22:6**	65.73	5.29	11	44.25	6.21	11	-32.7	0.0161	0.0357	No

Abbreviations: CON Control; **HD** Huntington’s disease; **PC** Phosphatidylcholine; **PD** Percentage Difference; **SEM** Standard Error of Mean.

Table S31 Ether PC Species in Control and HD White Cortex.

	CON Mean	SEM	N	HD Mean	SEM	N	PD (%)	p	q	Discovery?
**PC O-16:0_16:0**	120.31	7.33	11	109.66	6.03	11	-8.9	0.2754	0.2817	No
**PC O-16:1_16:0**	136.25	9.93	11	104.98	10.05	11	-23.0	0.0387	0.0684	No
**PC O-18:1_16:0**	1474.95	117.85	11	882.58	85.02	11	-40.2	0.0007	0.0033	Yes
**PC O-16:0_20:4**	76.84	4.74	11	47.72	6.06	11	-37.9	0.0010	0.0024	Yes
**PC O-16:1_20:4**	13.67	0.79	9	9.86	1.19	9	-27.9	0.0106	0.0130	No
**PC O-18:0_20:4**	80.34	6.00	11	49.41	4.80	7	-38.5	0.0012	0.0024	Yes
**PC O-18:1_20:4**	79.33	5.78	11	47.95	4.31	10	-39.6	0.0004	0.0031	Yes

Abbreviations: CON Control; **HD** Huntington's disease; **PC** Phosphatidylcholine; **PD** Percentage Difference; **SEM** Standard Error of Mean.

Table S32 Ester Linked PC Fatty Acids in Control and HD White Cortex.

	CON Mean	SEM	N	HD Mean	SEM	N	PD (%)	p	q	Discovery?
**16:0**	16968.87	892.37	11	12436.73	783.95	11	-26.7	0.0002	0.0005	Yes
**16:1**	5770.12	362.82	11	3845.07	279.99	11	-33.4	0.0005	0.0033	Yes
**18:0**	15350.62	783.12	11	10924.94	730.36	11	-28.8	0.0005	0.0033	Yes
**18:1**	31858.01	1636.29	11	22553.60	1441.65	11	-29.2	0.0001	0.0004	Yes
**18:2**	3041.02	131.15	11	1863.75	154.77	11	-38.7	0.0000	0.0005	Yes
**20:3**	184.29	8.41	11	132.81	15.14	11	-27.9	0.0092	0.0236	No
**20:4**	539.50	27.26	11	356.53	36.16	11	-33.9	0.0007	0.0033	Yes
**20:5**	85.11	4.01	11	55.81	9.61	11	-34.4	0.0143	0.0336	No
**22:4**	350.62	24.66	11	315.09	42.19	11	-10.1	0.4776	0.4188	No
**22:5**	179.40	26.73	11	113.35	17.37	11	-36.8	0.0536	0.0792	No
**22:6**	25654.29	1266.54	11	18334.66	1165.15	11	-28.5	0.0004	0.0031	Yes

Abbreviations: CON Control; **HD** Huntington's disease; **PC** Phosphatidylcholine; **PD** Percentage Difference; **SEM** Standard Error of Mean.

Table S33 Ether Linked PC Fatty Acids in Control and HD White Cortex.

	CON			HD						
	Mean	SEM	N	Mean	SEM	N	PD (%)	p	q	Discovery?
**O-16:0**	197.16	11.93	11	157.37	10.88	11	-20.2	0.0230	0.0448	No
**O-16:1**	147.43	10.43	11	113.04	10.51	11	-23.3	0.0336	0.0372	No
**O-18:0**	80.34	6.00	11	49.41	4.80	7	-38.5	0.0012	0.0024	Yes
**O-18:1**	1554.28	123.13	11	926.16	86.86	11	-40.4	0.0006	0.0033	Yes

Abbreviations: CON Control; **HD** Huntington's disease; **PC** Phosphatidylcholine; **PD** Percentage Difference; **SEM** Standard Error of Mean.

Table S34 Ester PE Species in Control and HD White Cortex.

	CON Mean	SEM	N	HD Mean	SEM	N	PD (%)	p	q	Discovery?
**PE 16:0_18:1**	381.13	26.57	12	424.66	34.95	12	11.4	0.3330	0.3201	No
**PE 16:1_18:0**	43.47	3.33	12	42.47	4.05	12	-2.3	0.8500	0.6584	No
**PE 16:0_18:2**	2.45	0.50	11	5.07	0.99	11	107.0	0.0329	0.0612	No
**PE 16:1_18:1**	99.84	8.45	12	95.89	8.85	12	-4.0	0.7497	0.6104	No
**PE 18:0_18:0**	5.21	0.45	12	5.25	0.68	10	0.8	0.9585	0.7225	No
**PE 18:0_18:1**	857.11	58.91	12	709.66	77.57	12	-17.2	0.1453	0.1633	No
**PE 18:0_18:2**	24.87	2.07	12	21.78	2.45	12	-12.4	0.3455	0.3282	No
**PE 18:1_18:1**	1296.48	94.20	12	1011.83	101.85	12	-22.0	0.0524	0.0788	No
**PE 16:0_20:3**	8.60	3.02	4	16.97	3.03	10	97.3	0.1878	0.1697	No
**PE 18:1_18:2**	84.54	5.72	12	77.73	5.78	12	-8.1	0.4114	0.3792	No
**PE 16:0_20:4**	88.31	6.47	12	87.76	8.47	12	-0.6	0.9598	0.7225	No
**PE 18:0_20:3**	163.37	14.85	12	158.23	15.77	12	-3.1	0.8148	0.6373	No
**PE 16:0_22:4**	111.47	8.74	12	103.21	10.10	12	-7.4	0.5428	0.4607	No
**PE 18:0_20:4**	650.57	52.08	12	619.61	87.28	12	-4.8	0.7642	0.6159	No
**PE 18:1_20:3**	81.28	6.24	12	75.50	9.15	12	-7.1	0.1600	0.1570	No
**PE 16:0_22:5**	157.66	13.27	12	150.38	11.24	12	-4.6	0.6797	0.5591	No
**PE 18:1_20:4**	295.72	21.03	12	259.73	28.41	12	-12.2	0.3206	0.3198	No
**PE 18:2_20:3**	2.39	0.15	10	2.03	0.50	7	-14.9	0.4747	0.3544	No
**PE 16:0_22:6**	120.17	15.08	12	129.09	30.85	12	7.4	0.7983	0.6307	No
**PE 16:1_22:5**	21.37	2.40	12	19.85	2.27	11	-7.1	0.6514	0.5414	No
**PE 18:1_20:5**	59.56	5.49	12	39.25	8.44	12	-34.1	0.0029	0.0056	Yes
**PE 18:2_20:4**	18.30	1.64	12	14.64	1.79	12	-20.0	0.1453	0.1633	No
**PE 18:0_22:4**	527.85	42.44	12	440.87	53.54	12	-16.5	0.2170	0.2404	No
**PE 18:0_22:5**	102.60	10.69	12	107.86	15.47	12	5.1	0.7824	0.6243	No
**PE 18:1_22:4**	302.58	26.97	12	226.05	24.66	12	-25.3	0.0481	0.0752	No
**PE 18:0_22:6**	1085.96	110.19	12	903.92	164.12	12	-16.8	0.3685	0.3459	No
**PE 18:1_22:5**	126.37	13.63	12	84.75	13.51	12	-32.9	0.0412	0.0684	No
**PE 18:1_22:6**	168.13	12.79	12	145.65	16.77	12	-13.4	0.1978	0.1698	No
**PE 18:2_22:6**	37.16	4.09	8	33.92	2.70	6	-8.7	0.6620	0.4639	No
**PE 20:3_22:6**	44.40	2.73	11	48.68	4.95	8	9.6	0.4640	0.4114	No

Abbreviations: CON Control; **HD** Huntington's disease; **PC** Phosphatidylcholine; **PD** Percentage Difference; **SEM** Standard Error of Mean.

Table S35 Ether PE Species in Control and HD White Cortex.

	CON			HD						
	Mean	SEM	N	Mean	SEM	N	PD (%)	p	q	Discovery?
**PE O-18:1_16:0**	703.79	71.01	12	516.39	50.42	12	-26.6	0.0439	0.0709	No
**PE O-16:1_18:1**	1244.02	120.74	12	900.12	125.74	12	-27.6	0.0612	0.0857	No
**PE O-18:2_16:0**	306.37	29.80	12	286.86	29.65	12	-6.4	0.9323	0.6157	No
**PE O-18:1_18:1**	1344.20	138.52	12	885.04	95.72	12	-34.2	0.0132	0.0318	No
**PE O-18:1_18:2**	3504.06	293.03	11	2430.69	308.55	10	-30.6	0.0208	0.0424	No
**PE O-16:1_20:3**	243.02	23.85	12	220.41	17.63	12	-9.3	0.4547	0.4111	No
**PE O-16:1_20:4**	163.65	16.96	12	150.50	17.74	12	-8.0	0.3186	0.2486	No
**PE O-18:0_20:4**	88.23	13.10	12	106.77	9.91	11	21.0	0.2604	0.2080	No
**PE O-18:1_20:3**	1044.57	123.49	12	763.42	77.70	12	-26.9	0.0694	0.0924	No
**PE O-16:1_22:4**	834.83	92.73	12	614.83	77.07	12	-26.4	0.0821	0.1032	No
**PE O-18:1_20:4**	227.99	23.03	12	193.83	18.08	12	-15.0	0.2565	0.2669	No
**PE O-18:2_20:3**	122.25	10.47	12	103.20	12.58	12	-15.6	0.2575	0.2669	No
**PE O-16:1_22:5**	65.84	8.39	12	62.28	6.05	12	-5.4	0.7987	0.5486	No
**PE O-18:2_20:4**	862.45	62.43	12	689.02	62.86	12	-20.1	0.0631	0.0868	No
**PE O-16:1_22:6**	134.28	12.37	12	124.68	8.17	11	-7.1	0.5251	0.4505	No
**PE O-18:1_22:4**	1164.51	117.37	12	899.05	98.19	12	-22.8	0.0972	0.1141	No
**PE O-18:0_22:6**	5.82	0.59	12	7.14	1.65	11	22.7	0.8801	0.5926	No
**PE O-18:1_22:5**	47.17	5.23	12	50.04	4.69	12	6.1	0.5899	0.4220	No
**PE O-18:2_22:4**	1270.05	98.70	12	924.56	96.26	12	-27.2	0.0201	0.0424	No
**PE O-18:1_22:6**	249.15	22.33	12	222.73	31.12	12	-10.6	0.4983	0.4322	No
**PE O-18:2_22:5**	92.95	8.31	12	85.91	11.65	12	-7.6	0.6281	0.5276	No
**PE O-18:2_22:6**	619.44	54.75	12	461.23	50.05	12	-25.5	0.0444	0.0709	No

Abbreviations: CON Control; **HD** Huntington's disease; **PD** Percentage Difference; **PE** Phosphatidylethanolamine; **SEM** Standard Error of Mean.

Table S36 Ester Linked PE Fatty Acids in Control and HD White Cortex.

	CON			HD						
	Mean	SEM	N	Mean	SEM	N	PD (%)	p	q	Discovery?
**16:0**	2369.03	176.67	12	2075.09	175.25	12	-12.4	0.2501	0.2669	No
**16:1**	67.01	5.61	12	48.26	8.61	12	-28.0	0.0083	0.0119	No
**18:0**	3762.13	266.04	12	3054.94	313.77	12	-18.8	0.0519	0.0557	No
**18:1**	1999.20	431.71	12	1417.39	201.79	12	-29.1	0.2189	0.1790	No
**18:2**	2173.35	152.29	12	1797.90	165.16	12	-17.3	0.1277	0.1290	No
**20:3**	1883.69	180.19	12	1547.45	122.35	12	-17.8	0.1782	0.1654	No
**20:4**	950.13	218.24	12	675.78	73.33	12	-28.9	0.5512	0.4027	No
**22:4**	2294.23	521.53	12	1631.39	263.16	12	-28.9	0.1782	0.1654	No
**22:5**	857.97	80.03	12	670.67	64.57	12	-21.8	0.0828	0.1032	No
**22:6**	1221.91	96.04	12	957.17	92.39	12	-21.7	0.0596	0.0849	No

Abbreviations: CON Control; **HD** Huntington's disease; **PD** Percentage Difference; **PE** Phosphatidylethanolamine; **SEM** Standard Error of Mean.

Table S37 Ether Linked PE Fatty Acids in Control and HD White Cortex.

	CON			HD						
	Mean	SEM	N	Mean	SEM	N	PD (%)	p	q	Discovery?
**O-16:1**	2685.64	264.73	12	2062.44	236.71	12	-23.2	0.0934	0.1129	No
**O-18:0**	94.05	13.22	12	113.91	10.74	11	21.1	0.2570	0.2669	No
**O-18:1**	7993.46	825.01	12	5556.07	678.91	12	-30.5	0.0330	0.0612	No
**O-18:2**	3273.49	250.95	12	2550.80	246.78	12	-22.1	0.0521	0.0788	No

Abbreviations: CON Control; **HD** Huntington's disease; **PD** Percentage Difference; **PE** Phosphatidylethanolamine; **SEM** Standard Error of Mean.

Table S38 PS Species in Control and HD White Cortex.

	CON Mean	SEM	N	HD Mean	SEM	N	PD (%)	p	q	Discovery?
**PS 16:0_18:1**	140.38	11.03	12	101.15	11.00	12	-27.9	0.0100	0.0128	No
**PS 18:0_18:1**	14390.06	1101.88	12	9143.56	1060.40	12	-36.5	0.0024	0.0071	Yes
**PS 18:0_18:2**	61.86	4.89	12	45.46	5.73	12	-26.5	0.0406	0.0684	No
**PS 18:1_18:1**	2515.69	213.83	12	1368.49	156.65	12	-45.6	0.0003	0.0031	Yes
**PS 18:0_20:3**	422.94	37.36	12	308.44	28.68	12	-27.1	0.0083	0.0119	No
**PS 18:0_20:4**	586.24	49.29	12	359.91	35.87	12	-38.6	0.0014	0.0050	Yes
**PS 18:1_20:4**	113.91	11.30	12	59.06	9.15	12	-48.1	0.0007	0.0016	Yes
**PS 18:0_22:4**	916.17	86.09	12	534.08	60.79	12	-41.7	0.0017	0.0055	Yes
**PS 18:0_22:5**	324.11	31.06	12	221.73	31.94	12	-31.6	0.0332	0.0372	No
**PS 18:0_22:6**	1052.28	91.17	12	879.70	146.74	12	-16.4	0.3308	0.3201	No

Abbreviations: CON Control; **HD** Huntington's disease; **PD** Percentage Difference; **PS** Phosphatidylserine; **SEM** Standard Error of Mean.

Table S39 PS Fatty Acids in Control and HD White Cortex.

	CON Mean	SEM	N	HD Mean	SEM	N	PD (%)	p	q	Discovery?
**16:0**	140.38	11.03	12	101.15	11.00	12	-27.9	0.0100	0.0128	No
**18:0**	17753.67	1335.46	12	11492.87	1204.87	12	-35.3	0.0021	0.0066	Yes
**18:1**	19675.74	1520.79	12	12040.75	1387.04	12	-38.8	0.0012	0.0047	Yes
**18:2**	61.86	4.89	12	45.46	5.73	12	-26.5	0.0406	0.0684	No
**20:3**	422.94	37.36	12	308.44	28.68	12	-27.1	0.0083	0.0119	No
**20:4**	700.14	60.20	12	418.98	44.42	12	-40.2	0.0012	0.0047	Yes
**22:4**	916.17	86.09	12	534.08	60.79	12	-41.7	0.0017	0.0055	Yes
**22:5**	324.11	31.06	12	221.73	31.94	12	-31.6	0.0332	0.0372	No
**22:6**	1052.28	91.17	12	879.70	146.74	12	-16.4	0.3308	0.3201	No

Abbreviations: CON Control; **HD** Huntington's disease; **PD** Percentage Difference; **PS** Phosphatidylserine; **SEM** Standard Error of Mean.

Table S40 Total Phospholipid Derived Ester Linked Fatty Acids in Control and HD White Cortex.

	CON			HD						
	Mean	SEM	N	Mean	SEM	N	PD (%)	p	q	Discovery?
**16:0**	19593.00	1041.96	11	13952.15	809.29	10	-28.8	0.0000	0.0000	Yes
**16:1**	5840.30	363.20	11	3782.46	289.18	10	-35.2	0.0003	0.0031	Yes
**18:0**	37856.78	2077.91	11	24102.13	1649.04	10	-36.3	0.0001	0.0010	Yes
**18:1**	54540.46	3226.88	11	34636.01	2348.59	10	-36.5	0.0000	0.0000	Yes
**18:2**	5384.16	249.34	11	3450.44	266.50	10	-35.9	0.0000	0.0010	Yes
**18:3**										
**20:3**	2599.01	210.38	11	1856.86	147.56	10	-28.6	0.0048	0.0082	Yes
**20:4**	2273.19	297.02	11	1367.46	132.94	10	-39.8	0.0004	0.0010	Yes
**20:5**	85.11	4.01	11	54.74	10.56	10	-35.7	0.0203	0.0424	No
**22:4**	3756.56	621.59	11	2440.68	387.81	10	-35.0	0.0610	0.0635	No
**22:5**	1424.36	120.46	11	954.02	101.88	10	-33.0	0.0101	0.0128	No
**22:6**	28055.98	1356.57	11	19518.49	1271.68	10	-30.4	0.0000	0.0000	Yes
**Total Saturated**	57449.78	3089.98	11	38054.28	2429.52	10	-33.8	0.0000	0.0000	Yes
**Total Monounsaturated**	60380.76	3571.91	11	38418.48	2627.50	10	-36.4	0.0000	0.0000	Yes
**Total Polyunsaturated**	43578.37	2740.21	11	29642.69	2179.99	10	-32.0	0.0000	0.0001	Yes
**Total Phospholipid**	161408.92	9289.05	11	106115.45	6997.98	10	-34.3	0.0000	0.0000	Yes

Abbreviations: CON Control; **HD** Huntington's disease; **PD** Percentage Difference; **SEM** Standard Error of Mean.

Table S41 Phospholipid Class Totals in Control and HD White Cortex.

	CON			HD						
	Mean	SEM	N	Mean	SEM	N	PD (%)	p	q	Discovery?
**Total Ester PC**	25402.41	1254.05	11	18182.46	1155.50	11	-28.4	0.0001	0.0003	Yes
**Total Ether PC**	1979.21	148.62	11	1228.02	104.97	11	-38.0	0.0006	0.0033	Yes
**Total Ester PE**	6946.47	480.46	12	6022.46	596.55	12	-13.3	0.2411	0.2635	No
**Total Ether PE**	14046.65	1311.70	12	10273.73	1127.64	12	-26.9	0.0404	0.0684	No
**Total PS (Ester)**	20523.65	1554.27	12	13021.57	1375.81	12	-36.6	0.0016	0.0054	Yes

Abbreviations: CON Control; **HD** Huntington's disease; **PC** Phosphatidylcholine; **PD** Percentage Difference; **PE** Phosphatidylethanolamine; **PS** Phosphatidylserine; **SEM** Standard Error of Mean.

Table S42 LPC and LPE in Control and HD White Cortex.

White	CON Mean	SEM	N	HD Mean	SEM	N	PD (%)	p	q	Discovery?
**LPC 16:0**	36.31	4.38	12	24.06	2.81	13	-33.74	0.02	0.04	No
**LPC 16:1**	2.81	0.35	12	1.85	0.26	13	-34.17	0.03	0.04	No
**LPC 18:0**	19.82	2.15	12	11.31	1.52	13	-42.91	0.00	0.04	No
**LPC 18:1**	53.63	5.12	12	34.25	4.13	13	-36.14	0.01	0.04	No
**LPC 18:2**	1.52	0.22	12	0.86	0.17	13	-43.33	0.02	0.04	No
**LPC 20:4**	5.27	0.42	12	3.97	0.37	13	-24.63	0.03	0.07	No
**LPC 22:4**	2.99	0.45	12	1.69	0.26	13	-43.24	0.02	0.06	No
**LPC 22:6**	2.51	0.34	12	1.39	0.25	13	-44.65	0.02	0.06	No
**Total LPC**	124.85	12.68	12	79.38	9.14	13	-36.41	0.00	0.02	No
**LPE 16:0**	65.05	11.53	12	52.01	8.68	13	-20.06	0.38	0.42	No
**LPE 16:1**	16.09	2.53	12	13.34	2.55	13	-17.10	0.45	0.46	No
**LPE 18:0**	41.32	12.37	12	21.69	3.56	13	-47.51	0.07	0.08	No
**LPE 18:1**	934.66	127.81	12	575.88	121.42	13	-38.39	0.05	0.09	No
**LPE 18:2**	6.74	1.06	11	5.06	0.81	9	-24.92	0.49	0.50	No
**LPE 22:5**	25.04	3.70	12	20.26	3.20	13	-19.09	0.34	0.42	No
**LPE 22:6**	65.33	10.88	12	43.11	5.41	13	-34.02	0.09	0.12	No
**Total LPE**	1153.67	158.31	12	729.78	138.85	13	-36.74	0.06	0.09	No

Abbreviations: CON Control; **HD** Huntington’s disease; **LPC** Lysophosphatidylcholine; **LPE** Lysophospatidylethanolamine; **PD** Percentage Difference; **SEM** Standard Error of Mean.

3.3.4 Grey Matter of the Dorsomedial Prefrontal Cortex

Table S43 Ester PC Species in Control and HD Grey Cortex.

	CON			HD						
	Mean	SEM	N	Mean	SEM	N	PD (%)	p	q	Discovery?
**PC 16:0_16:0**	2910.53	171.59	13	2526.46	134.83	13	-13.20	0.1534	0.4184	No
**PC 16:0_16:1**	534.83	42.26	13	453.45	43.08	13	-15.22	0.1690	0.4184	No
**PC 16:0_18:0**	555.25	50.13	13	452.85	36.53	13	-18.44	0.1130	0.2563	No
**PC 16:0_18:1**	8623.82	517.53	13	7006.18	532.75	13	-18.76	0.0395	0.1561	No
**PC 16:1_18:0**	111.30	8.02	13	94.16	9.24	13	-15.40	0.1740	0.3292	No
**PC 16:0_18:2**	167.66	11.64	13	174.60	15.78	13	4.14	0.7267	0.8669	No
**PC 16:1_18:1**	198.03	17.80	13	164.49	16.51	13	-16.94	0.1800	0.3321	No
**PC 16:0_18:3**	21.48	2.44	10	20.49	2.29	12	-4.62	0.7700	0.8805	No
**PC 18:0_18:1**	2180.23	210.97	13	1661.46	214.07	13	-23.79	0.0972	0.2402	No
**PC 18:0_18:2**	68.38	5.39	13	65.27	6.29	13	-4.55	0.7104	0.8669	No
**PC 18:1_18:1**	801.16	107.95	13	565.76	70.94	13	-29.38	0.1014	0.4184	No
**PC 16:0_20:3**	133.18	6.42	13	127.39	9.86	13	-4.35	0.6275	0.7894	No
**PC 18:1_18:2**	60.70	6.70	13	52.12	4.11	13	-14.14	0.2879	0.4687	No
**PC 16:0_20:4**	713.06	36.33	13	621.73	34.95	13	-12.81	0.0826	0.2198	No
**PC 16:0_20:5**	10.27	2.61	10	8.81	1.71	11	-14.17	0.6479	0.8076	No
**PC 16:1_20:4**	30.89	2.33	13	24.31	1.98	13	-21.30	0.0417	0.1603	No
**PC 18:0_20:3**	67.26	3.79	13	64.65	6.31	13	-3.89	0.7264	0.8669	No
**PC 16:0_22:4**	109.78	4.91	13	94.31	6.11	13	-14.09	0.0605	0.1868	No
**PC 18:0_20:4**	691.05	35.44	13	578.00	30.10	13	-16.36	0.0231	0.1305	No
**PC 18:1_20:3**	64.46	7.58	13	50.20	6.32	13	-22.12	0.1690	0.4184	No
**PC 16:0_22:5**	27.44	2.37	13	32.39	2.83	13	18.04	0.2428	0.4444	No
**PC 18:0_20:5**	79.02	13.75	13	60.89	14.47	13	-22.94	0.3729	0.5549	No
**PC 18:1_20:4**	137.93	9.45	13	119.69	8.43	13	-13.22	0.1627	0.3128	No
**PC 16:0_22:6**	395.34	35.17	13	313.07	22.35	13	-20.81	0.0621	0.1868	No
**PC 18:1_20:5**	46.47	11.72	13	30.01	7.78	13	-35.41	0.3358	0.5089	No
**PC 18:2_20:4**	7.74	0.82	10	7.64	0.92	13	-1.26	0.9378	0.9684	No
**PC 16:1_22:6**	29.94	1.46	13	24.11	1.29	13	-19.47	0.0063	0.0832	No
**PC 18:0_22:4**	100.55	3.86	13	87.29	4.25	13	-13.18	0.0300	0.1384	No
**PC 18:0_22:5**	53.71	2.90	12	51.34	3.16	13	-4.40	0.5871	0.7664	No

Table S43 Continued… Ester PC Species in Control and HD Grey Cortex.

	CON			HD						
	**Mean**	**SEM**	**N**	**Mean**	**SEM**	**N**	**PD (%)**	**p**	**q**	**Discovery?**
**PC 18:0_22:6**	254.86	22.98	13	190.84	11.69	13	-25.12	0.0232	0.1305	No
**PC 18:1_22:5**	27.20	4.09	13	21.36	2.38	13	-21.48	0.2313	0.4000	No
**PC 18:1_22:6**	115.55	7.72	13	84.26	4.88	13	-27.08	0.0026	0.0832	No
**PC 18:2_22:6**	9.52	1.45	10	10.04	0.52	11	5.46	0.7422	0.8777	No

Abbreviations: CON Control; **HD** Huntington's disease; **PC** Phosphatidylcholine; **PD** Percentage Difference; **SEM** Standard Error of Mean.

Table S44 Ether PC Species Control and HD Grey Cortex.

	CON			HD						
	**Mean**	**SEM**	**N**	**Mean**	**SEM**	**N**	**PD (%)**	**p**	**q**	**Discovery?**
**PC O-16:0_16:0**	90.86	5.29	13	92.67	7.52	13	1.99	0.8461	0.9345	No
**PC O-16:1_16:0**	39.61	4.71	13	37.55	5.23	13	-5.22	0.5446	0.7596	No
**PC O-18:1_16:0**	333.67	52.01	13	264.65	43.75	13	-20.69	0.2035	0.4258	No
**PC O-16:0_20:4**	29.97	4.10	13	24.47	3.12	13	-18.36	0.2972	0.4727	No
**PC O-16:1_20:4**	9.97	0.90	10	11.40	1.77	13	14.35	0.8793	0.9198	No
**PC O-18:0_20:4**	35.21	2.70	12	32.39	2.26	13	-8.01	0.4316	0.5972	No
**PC O-18:1_20:4**	29.58	2.61	12	27.53	2.61	13	-6.93	0.5843	0.7664	No

Abbreviations: CON Control; **HD** Huntington's disease; **PC** Phosphatidylcholine; **PD** Percentage Difference; **SEM** Standard Error of Mean.

Table S45 Ester Linked PC Fatty Acids in Control and HD Grey Cortex.

	CON			HD						
	**Mean**	**SEM**	**N**	**Mean**	**SEM**	**N**	**PD (%)**	**p**	**q**	**Discovery?**
**16:0**	17569.98	934.51	13	14750.12	855.16	13	-16.05	0.0357	0.1498	No
**16:1**	904.99	69.42	13	760.53	69.87	13	-15.96	0.1554	0.3128	No
**18:0**	4157.47	236.29	13	3306.74	255.05	13	-20.46	0.0222	0.1305	No
**18:1**	13056.71	945.03	13	10321.30	907.31	13	-20.95	0.0476	0.1661	No
**18:2**	310.02	22.22	13	308.12	24.60	13	-0.61	0.9549	0.9788	No
**18:3**	21.48	2.44	10	20.49	2.29	12	-4.62	0.7700	0.8805	No
**20:3**	264.91	12.01	13	242.24	16.05	13	-8.56	0.2702	0.4504	No
**20:4**	1676.33	82.18	13	1447.16	74.91	13	-13.67	0.0504	0.1661	No
**20:5**	133.38	24.42	13	98.36	21.63	13	-26.26	0.1857	0.4184	No
**22:4**	210.32	8.13	13	181.60	9.97	13	-13.66	0.0355	0.1498	No
**22:5**	104.21	7.16	13	105.09	5.50	13	0.84	0.9236	0.9677	No
**22:6**	803.01	65.64	13	620.77	37.62	13	-22.69	0.0262	0.1358	No

Abbreviations: CON Control; **HD** Huntington's disease; **PC** Phosphatidylcholine; **PD** Percentage Difference; **SEM** Standard Error of Mean.

Table S46Ether Linked PC Fatty Acids in Control and HD Grey Cortex.

	CON			HD						
	**Mean**	**SEM**	**N**	**Mean**	**SEM**	**N**	**PD (%)**	**p**	**q**	**Discovery?**
**O-16:0**	120.83	8.60	13	117.13	9.87	13	-3.06	0.7802	0.8849	No
**O-16:1**	47.28	4.45	13	48.95	5.33	13	3.52	0.8126	0.9068	No
**O-18:0**	35.21	2.70	12	32.39	2.26	13	-8.01	0.4316	0.5972	No
**O-18:1**	360.97	51.41	13	292.18	46.20	13	-19.06	0.1857	0.4184	No

Abbreviations: CON Control; **HD** Huntington’s disease; **PC** Phosphatidylcholine; **PD** Percentage Difference; **SEM** Standard Error of Mean.

Table S47 Ester PE Species in Control and HD Grey Cortex.

	CON Mean	SEM	N	HD Mean	SEM	N	PD (%)	p	q	Discovery?
**PE 16:0_18:1**	388.62	17.57	13	307.98	20.58	13	-20.75	0.0066	0.0832	No
**PE 16:1_18:0**	39.70	3.06	13	35.70	3.15	13	-10.07	0.3714	0.5549	No
**PE 16:0_18:2**	7.71	1.13	12	9.16	0.87	13	18.87	0.3173	0.4989	No
**PE 16:1_18:1**	56.76	6.12	13	44.33	5.37	13	-21.90	0.1399	0.2934	No
**PE 18:0_18:0**	7.83	0.50	13	7.67	0.64	12	-2.06	0.3475	0.5089	No
**PE 18:0_18:1**	523.18	37.09	13	395.52	33.48	13	-24.40	0.0175	0.1305	No
**PE 18:0_18:2**	37.56	3.51	13	37.71	3.47	13	0.41	0.9753	0.9851	No
**PE 18:1_18:1**	487.51	56.66	13	338.86	50.15	13	-30.49	0.0613	0.1868	No
**PE 16:0_20:3**	29.06	2.83	13	31.98	4.96	13	10.05	0.6148	0.7805	No
**PE 18:1_18:2**	45.93	6.85	13	39.10	4.05	13	-14.88	0.4007	0.5899	No
**PE 16:0_20:4**	146.95	8.89	13	122.02	8.17	13	-16.96	0.0500	0.1661	No
**PE 18:0_20:3**	194.16	10.18	13	179.68	16.22	13	-7.46	0.4584	0.6280	No
**PE 16:0_22:4**	135.76	6.12	13	107.59	7.14	13	-20.75	0.0064	0.0832	No
**PE 18:0_20:4**	1560.28	73.76	13	1297.19	102.90	13	-16.86	0.0497	0.1661	No
**PE 18:1_20:3**	51.33	4.45	13	48.52	6.05	13	-5.48	0.7113	0.8669	No
**PE 16:0_22:5**	136.99	7.55	13	125.04	10.59	13	-8.72	0.3683	0.5549	No
**PE 18:0_20:5**	5.80	0.89	9	2.84	0.42	10	-51.08	0.0030	0.0617	No
**PE 18:1_20:4**	228.16	17.64	13	180.20	15.55	13	-21.02	0.0528	0.1697	No
**PE 16:0_22:6**	601.60	44.47	13	456.32	35.34	13	-24.15	0.0176	0.1305	No
**PE 16:1_22:5**	17.93	2.33	12	16.26	2.21	13	-9.30	0.7689	0.9008	No
**PE 18:1_20:5**	22.23	2.92	13	17.26	3.47	13	-22.38	0.1857	0.4184	No
**PE 18:2_20:4**	15.19	1.66	13	13.14	1.32	13	-13.49	0.3444	0.5295	No
**PE 16:1_22:6**	24.89	2.25	8	19.88	1.14	9	-20.11	0.0741	0.2052	No
**PE 18:0_22:4**	923.72	36.24	13	735.84	45.81	13	-20.34	0.0039	0.0832	No
**PE 18:0_22:5**	303.58	18.19	13	314.75	39.95	13	3.68	0.8021	0.9023	No
**PE 18:1_22:4**	144.71	14.59	13	109.34	11.37	13	-24.44	0.0685	0.1938	No
**PE 18:0_22:6**	3787.54	282.15	13	2680.43	180.75	13	-29.23	0.0035	0.0832	No
**PE 18:1_22:5**	72.91	6.21	13	65.75	5.34	13	-9.82	0.6139	0.7818	No
**PE 18:1_22:6**	303.68	16.37	13	243.29	16.30	13	-19.88	0.0152	0.1305	No
**PE 18:2_22:6**	29.62	4.15	10	26.23	1.97	9	-11.45	0.8421	0.9198	No
**PE 20:4_22:6**	25.94	1.07	9	21.31	2.47	7	-17.84	0.0712	0.4184	No

Table S47 Continued… Ester PE Species in Control and HD Grey Cortex.

	CON Mean	SEM	N	HD Mean	SEM	N	PD (%)	p	q	Discovery?
**PE 20:3_22:6**	101.81	7.38	13	75.46	7.12	13	-25.88	0.0042	0.0617	No

Abbreviations: CON Control; **HD** Huntington's disease; **PD** Percentage Difference; **PE** Phosphatidylethanolamine; **SEM** Standard Error of Mean.

Table S48 Ether PE Species in Control and HD Grey Cortex.

	CON Mean	SEM	N	HD Mean	SEM	N	PD (%)	p	q	Discovery?
**PE O-18:1_16:0**	219.53	25.46	13	162.40	18.68	12	-26.02	0.0844	0.2203	No
**PE O-16:1_18:1**	225.21	48.87	12	190.29	45.35	9	-15.50	0.6066	0.7772	No
**PE O-18:2_16:0**	78.62	13.62	12	75.04	14.55	9	-4.56	0.8621	0.9198	No
**PE O-18:1_18:1**	280.70	44.02	12	182.35	32.63	12	-35.04	0.1005	0.4184	No
**PE O-18:1_18:2**	519.04	97.68	11	429.63	110.42	11	-17.23	0.5511	0.7476	No
**PE O-16:1_20:3**	145.74	12.96	12	115.65	8.42	11	-20.65	0.0667	0.1938	No
**PE O-16:1_20:4**	83.50	5.76	11	82.83	4.83	9	-0.80	0.9301	0.9677	No
**PE O-18:0_20:4**	83.62	15.57	11	62.03	10.47	11	-25.81	0.2654	0.4478	No
**PE O-18:1_20:3**	495.15	39.64	13	412.09	27.50	13	-16.77	0.0996	0.2413	No
**PE O-16:1_22:4**	211.84	30.19	13	159.31	23.40	13	-24.80	0.1826	0.3324	No
**PE O-18:1_20:4**	301.33	12.72	13	259.95	15.45	13	-13.73	0.0500	0.1661	No
**PE O-18:2_20:3**	36.51	6.90	13	30.69	7.17	13	-15.95	0.5637	0.7500	No
**PE O-16:1_22:5**	46.40	3.33	13	46.97	3.07	13	1.23	0.9008	0.9644	No
**PE O-18:1_20:5**	10.03	2.87	10	7.69	1.35	13	-23.31	0.9274	0.9366	No
**PE O-18:2_20:4**	500.95	45.15	13	408.92	34.41	13	-18.37	0.1190	0.2588	No
**PE O-16:1_22:6**	138.72	10.33	13	128.30	12.94	13	-7.51	0.5788	0.7706	No
**PE O-18:1_22:4**	558.17	44.68	13	464.82	41.35	13	-16.72	0.1384	0.2934	No
**PE O-18:0_22:6**	50.17	6.85	13	53.78	9.59	13	7.21	0.7618	0.8805	No
**PE O-18:1_22:5**	153.42	16.67	13	173.63	17.49	13	13.17	0.4112	0.5918	No
**PE O-18:2_22:4**	615.34	67.36	13	429.42	49.92	13	-30.21	0.0372	0.1513	No
**PE O-18:1_22:6**	521.61	27.73	13	440.19	32.42	13	-15.61	0.0686	0.1938	No
**PE O-18:2_22:5**	23.98	3.35	13	23.78	4.66	13	-0.82	0.7623	0.9008	No
**PE O-18:2_22:6**	246.93	27.22	13	206.15	15.57	12	-16.52	0.2090	0.3756	No

Abbreviations: CON Control; **HD** Huntington's disease; **PD** Percentage Difference; **PE** Phosphatidylethanolamine; **SEM** Standard Error of Mean.

Table S49 Ester Linked PE Fatty Acids in Control and HD Grey Cortex.

	CON			HD						
	Mean	SEM	N	Mean	SEM	N	PD (%)	p	q	Discovery?
**16:0**	1738.19	82.18	13	1361.95	92.13	13	-21.65	0.0056	0.0832	No
**16:1**	128.33	12.19	13	110.06	11.99	13	-14.24	0.2958	0.4727	No
**18:0**	7389.40	389.28	13	5693.18	353.85	13	-22.95	0.0036	0.0832	No
**18:1**	3279.52	306.81	13	2429.07	266.48	13	-25.93	0.0473	0.1661	No
**20:3**	1042.55	70.24	13	876.28	68.42	13	-15.95	0.1029	0.2413	No
**20:4**	2912.23	131.60	13	2402.74	169.55	13	-17.50	0.0265	0.1358	No
**20:5**	33.96	4.43	13	27.13	3.23	13	-20.11	0.2226	0.4346	No
**22:4**	2589.54	165.38	13	2006.34	153.72	13	-22.52	0.0164	0.1305	No
**22:5**	753.81	31.69	13	766.17	56.62	13	1.64	0.8509	0.9345	No
**22:6**	5808.12	367.53	13	4311.47	293.65	13	-25.77	0.0042	0.0832	No

Abbreviations: CON Control; **HD** Huntington's disease; **PD** Percentage Difference; **PE** Phosphatidylethanolamine; **SEM** Standard Error of Mean.

Table S50 Ether Linked Fatty Acids in Control and HD Grey Cortex.

	CON			HD						
	Mean	SEM	N	Mean	SEM	N	PD (%)	p	q	Discovery?
**O-16:1**	810.02	104.13	13	621.52	86.02	13	-23.27	0.1761	0.3292	No
**O-18:0**	120.92	19.15	13	106.27	15.84	13	-12.11	0.5613	0.7500	No
**O-18:1**	2955.22	218.64	13	2440.14	217.80	13	-17.43	0.1081	0.2493	No
**O-18:2**	1496.29	154.51	13	1135.06	122.72	13	-24.14	0.0802	0.2177	No

Abbreviations: CON Control; **HD** Huntington's disease; **PD** Percentage Difference; **PE** Phosphatidylethanolamine; **SEM** Standard Error of Mean.

Table S51 PS Species in Control and HD Grey Cortex.

	CON Mean	SEM	N	HD Mean	SEM	N	PD (%)	p	q	Discovery?
**PS 16:0_18:1**	141.91	17.75	13	113.22	5.83	11	-20.21	0.2767	0.4502	No
**PS 18:0_18:1**	2886.06	572.13	13	2021.07	527.33	13	-29.97	0.2773	0.4569	No
**PS 18:0_18:2**	18.75	2.59	13	19.30	3.07	13	2.92	0.8930	0.9644	No
**PS 18:1_18:1**	560.53	98.60	13	396.32	85.56	13	-29.30	0.1129	0.4184	No
**PS 18:0_20:3**	158.44	15.88	13	161.44	21.99	12	1.89	0.9130	0.9644	No
**PS 18:0_20:4**	231.18	25.48	13	201.39	25.20	11	-12.88	0.4149	0.5918	No
**PS 18:0_22:4**	508.36	31.34	13	436.56	31.60	13	-14.12	0.1197	0.2588	No
**PS 18:0_22:5**	253.29	25.03	13	282.51	48.34	13	11.54	0.5979	0.7732	No
**PS 18:0_22:6**	2635.39	174.25	13	2300.28	198.71	13	-12.72	0.2172	0.3804	No

Abbreviations: CON Control; **HD** Huntington's disease; **PD** Percentage Difference; **PS** Phosphatidylserine; **SEM** Standard Error of Mean.

Table S52 PS Fatty Acids in Control and HD Grey Cortex.

	CON Mean	SEM	N	HD Mean	SEM	N	PD (%)	p	q	Discovery?
**16:0**	141.91	17.75	13	113.22	5.83	11	-20.21	0.2767	0.4502	No
**18:0**	6691.47	652.46	13	5379.14	626.67	13	-19.61	0.1599	0.3128	No
**18:1**	4149.04	767.25	13	2909.51	700.24	13	-29.87	0.1389	0.4184	No
**18:2**	18.75	2.59	13	19.30	3.07	13	2.92	0.8930	0.9644	No
**20:3**	158.44	15.88	13	161.44	21.99	12	1.89	0.9130	0.9644	No
**20:4**	231.18	25.48	13	201.39	25.20	11	-12.88	0.4149	0.5918	No
**22:4**	508.36	31.34	13	436.56	31.60	13	-14.12	0.1197	0.2588	No
**22:5**	253.29	25.03	13	306.05	45.89	12	20.83	0.3268	0.5081	No
**22:6**	2635.39	174.25	13	2300.28	198.71	13	-12.72	0.2172	0.3804	No

Abbreviations: CON Control; **HD** Huntington's disease; **PD** Percentage Difference; **PS** Phosphatidylserine; **SEM** Standard Error of Mean.

Table S53 Total Phospholipid Derived Ester Linked Fatty Acids in Control and HD Grey Cortex.

	CON			HD						
	Mean	SEM	N	Mean	SEM	N	PD (%)	p	q	Discovery?
**16:0**	19450.08	1011.86	13	16207.88	946.69	13	-16.67	0.0280	0.1360	No
**16:1**	1033.32	77.89	13	870.59	81.32	13	-15.75	0.1613	0.3128	No
**18:0**	18238.34	978.46	13	14379.06	1069.02	13	-21.16	0.0137	0.1305	No
**18:1**	20485.26	1967.24	13	15659.89	1841.17	13	-23.56	0.0860	0.2203	No
**18:2**	328.77	24.02	13	327.42	26.75	13	-0.41	0.9704	0.9851	No
**18:3**	21.48	2.44	10	20.49	2.29	12	-4.62	0.7700	0.8805	No
**20:3**	1465.89	93.79	13	1267.54	100.30	13	-13.53	0.1616	0.3128	No
**20:4**	4819.74	208.80	13	4020.31	255.29	13	-16.59	0.0236	0.1305	No
**20:5**	167.35	26.12	13	125.49	24.35	13	-25.01	0.2528	0.4318	No
**22:4**	3308.22	200.29	13	2624.49	189.15	13	-20.67	0.0205	0.1305	No
**22:5**	1111.31	55.70	13	1153.77	105.42	13	3.82	0.7258	0.8669	No
**22:6**	9246.52	589.38	13	7232.52	503.86	13	-21.78	0.0160	0.1305	No
**Total Saturated**	37688.42	1959.12	13	30586.94	2004.02	13	-18.84	0.0182	0.1305	No
**Total Monounsaturated**	21518.59	2041.65	13	16530.47	1918.10	13	-23.18	0.0877	0.2206	No
**Total Polyunsaturated**	20464.33	899.03	13	16770.45	1013.25	13	-18.05	0.0118	0.1305	No
**Total Phospholipid**	79671.34	4465.13	13	63887.86	4519.45	13	-19.81	0.0204	0.1305	No

Abbreviations: CON Control; **HD** Huntington's disease; **PD** Percentage Difference; **SEM** Standard Error of Mean.

Table S54 Phospholipid Class Totals in Control and HD Grey Cortex.

	CON			HD						
	Mean	SEM	N	Mean	SEM	N	PD (%)	p	q	Discovery?
**Total Ester PC**	19323.14	1077.29	13	15835.15	1038.53	13	-18.05	0.0285	0.1360	No
**Total Ether PC**	561.59	62.31	13	490.65	61.45	13	-12.63	0.4255	0.5972	No
**Total Ester PE**	10430.49	491.76	13	8081.10	489.24	13	-22.52	0.0024	0.0832	No
**Total Ether PE**	5382.45	467.61	13	4303.00	427.34	13	-20.06	0.1014	0.2413	No
**Total PS (Ester)**	7393.91	747.16	13	5871.27	711.59	13	-20.59	0.1530	0.3128	No

Abbreviations: CON Control; **HD** Huntington's disease; **PC** Phosphatidylcholine; **PD** Percentage Difference; **PE** Phosphatidylethanolamine; **PS** Phosphatidylserine; **SEM** Standard Error of Mean.

Table S55 LPC and LPE Species in Control and HD Grey Cortex.

Grey	CON Mean	SEM	N	HD Mean	SEM	N	PD (%)	p	q	Discovery?
**LPC 16:0**	34.05	1.59	13	28.89	2.61	13	-15.14	0.04	0.07	No
**LPC 16:1**	2.65	0.24	13	1.61	0.27	13	-39.20	0.01	0.05	No
**LPC 18:0**	13.04	0.62	13	10.56	0.94	13	-19.02	0.04	0.06	No
**LPC 18:1**	35.11	1.69	13	25.88	2.23	13	-26.30	0.00	0.02	No
**LPC 18:2**	1.59	0.12	13	1.28	0.25	13	-19.26	0.03	0.07	No
**LPC 20:4**	7.53	0.53	13	5.66	0.35	13	-24.79	0.01	0.03	No
**LPC 22:4**	2.09	0.19	13	1.65	0.30	13	-21.40	0.22	0.27	No
**LPC 22:6**	4.79	0.48	13	3.35	0.23	13	-30.09	0.01	0.03	No
**Total LPC**	100.85	4.71	13	78.88	6.40	13	-21.78	0.01	0.03	No
**LPE 16:0**	27.65	3.29	13	20.61	5.35	13	-25.44	0.28	0.31	No
**LPE 16:1**	6.41	1.61	13	5.83	1.92	13	-8.97	0.76	0.77	No
**LPE 18:0**	48.31	2.36	13	32.33	2.91	13	-33.07	0.00	0.00	Yes
**LPE 18:1**	196.04	29.89	13	167.10	51.68	13	-14.76	0.63	0.58	No
**LPE 22:5**	27.89	3.80	12	22.73	4.28	12	-18.50	0.38	0.38	No
**LPE 22:6**	206.25	36.42	13	113.26	21.86	13	-45.08	0.04	0.06	No
**Total LPE**	510.41	66.15	13	360.13	80.77	13	-29.44	0.11	0.14	No

Abbreviations: CON Control; **HD** Huntington's disease; **LPC** Lysophosphatidylcholine; **LPE** Lysophopshatidylethanolamine; **PD** Percentage Difference; **SEM** Standard Error of Mean.

3.3.5 Cerebellum

Table S56 Ester PC Species in Control and HD Cerebellum.

	CON Mean	SEM	N	HD Mean	SEM	N	PD (%)	p	q	Discovery?
**PC 16:0_16:0**	2254.20	204.00	12	2185.18	144.48	13	-3.06	0.7853	0.9538	No
**PC 16:0_16:1**	364.18	40.48	12	416.72	29.46	13	14.43	0.3063	0.9345	No
**PC 16:0_18:0**	575.44	55.93	12	414.27	33.84	13	-28.01	0.0238	0.6945	No
**PC 16:0_18:1**	5409.00	541.03	12	5757.38	462.84	13	6.44	0.6294	0.9345	No
**PC 16:1_18:0**	47.21	4.53	12	51.83	4.61	13	9.80	0.4812	0.9345	No
**PC 16:0_18:2**	137.68	12.76	12	174.87	22.23	13	27.01	0.3475	0.8858	No
**PC 16:1_18:1**	115.50	10.68	12	151.41	22.17	13	31.10	0.3760	0.9149	No
**PC 16:0_18:3**	14.72	2.22	11	13.34	1.18	13	-9.40	0.5905	0.9345	No
**PC 18:0_18:1**	1024.89	102.56	12	1089.97	123.17	13	6.35	0.6885	0.9345	No
**PC 18:0_18:2**	43.54	4.24	12	46.24	7.17	13	6.22	0.7487	0.9345	No
**PC 18:1_18:1**	450.48	46.51	12	510.88	55.39	13	13.41	0.4125	0.9345	No
**PC 16:0_20:3**	90.06	9.76	12	88.04	4.66	13	-2.24	0.8545	0.9779	No
**PC 18:1_18:2**	41.75	4.14	12	60.76	15.14	13	45.52	0.4696	0.9937	No
**PC 16:0_20:4**	726.73	63.71	12	708.75	42.80	13	-2.47	0.8172	0.9779	No
**PC 16:0_20:5**	10.74	1.89	12	10.68	1.82	13	-0.53	0.9829	>0.9999	No
**PC 16:1_20:4**	17.24	1.67	12	18.34	1.93	13	6.36	0.6713	0.9345	No
**PC 18:0_20:3**	51.85	5.93	12	53.38	6.65	13	2.96	0.8100	>0.9999	No
**PC 16:0_22:4**	48.11	5.38	12	46.93	7.34	13	-2.47	0.9787	>0.9999	No
**PC 18:0_20:4**	530.20	45.46	12	444.56	31.39	13	-16.15	0.1369	0.8978	No
**PC 18:1_20:3**	32.91	4.58	12	36.69	8.82	13	11.49	0.9362	>0.9999	No
**PC 16:0_22:5**	29.70	3.70	12	30.74	5.12	13	3.51	0.8704	0.9779	No
**PC 18:0_20:5**	31.89	4.45	12	45.37	8.26	13	42.28	0.1676	0.8978	No
**PC 18:1_20:4**	155.22	14.59	12	130.06	7.10	13	-16.21	0.1405	0.8978	No
**PC 16:0_22:6**	475.04	47.05	12	399.90	32.04	13	-15.82	0.2019	0.8978	No
**PC 18:1_20:5**	25.15	3.72	12	31.20	4.67	13	24.02	0.3221	0.9345	No
**PC 18:2_20:4**	4.46	1.28	10	3.83	0.97	10	-14.27	0.8534	>0.9999	No
**PC 16:1_22:6**	24.12	1.95	12	23.09	0.99	12	-4.28	0.6438	0.9345	No
**PC 18:0_22:4**	37.19	3.35	12	45.58	3.65	13	22.56	0.1038	0.8978	No
**PC 18:0_22:5**	44.83	4.28	12	47.75	3.47	13	6.52	0.6114	0.9937	No

Table S56 Continued… Ester PC Species in Control and HD Cerebellum.

	CON			HD						
	**Mean**	**SEM**	**N**	**Mean**	**SEM**	**N**	**PD (%)**	**p**	**q**	**Discovery?**
**PC 18:0_22:6**	319.34	30.89	12	251.85	22.37	13	-21.13	0.0918	0.8978	No
**PC 18:1_22:5**	13.01	2.38	12	15.44	2.50	12	18.64	0.4905	0.9345	No
**PC 18:1_22:6**	133.57	12.02	12	110.84	8.65	13	-17.02	0.1402	0.8978	No
**PC 18:2_22:6**	10.65	1.33	9	9.71	0.94	12	-8.85	0.5719	0.9345	No

Abbreviations: CON Control; **HD** Huntington's disease; **PC** Phosphatidylcholine; **PD** Percentage Difference; **SEM** Standard Error of Mean.

Table S57 Ether PC Species in Control and HD Cerebellum.

	CON Mean	SEM	N	HD Mean	SEM	N	PD (%)	p	q	Discovery?
**PC O-16:0_16:0**	91.79	7.89	12	106.86	6.53	13	16.42	0.0868	0.4222	No
**PC O-16:1_16:0**	18.39	1.12	12	28.82	6.42	13	56.75	0.0868	0.4222	No
**PC O-18:1_16:0**	162.85	17.74	12	214.07	25.75	13	31.45	0.0976	0.4356	No
**PC O-18:0_18:2**	32.81	4.89	9	47.37	7.38	13	44.36	0.1164	0.8978	No
**PC O-16:0_20:4**	16.15	1.38	12	23.22	4.50	13	43.77	0.0678	0.4222	No
**PC O-16:1_20:4**	13.89	3.13	12	19.76	6.32	13	42.34	0.6495	0.9997	No
**PC O-18:0_20:4**	24.13	2.13	12	28.85	2.14	13	19.59	0.1311	0.8978	No
**PC O-18:1_20:4**	17.19	1.64	12	25.12	4.22	12	46.14	0.0284	0.4222	No

Abbreviations: CON Control; **HD** Huntington's disease; **PC** Phosphatidylcholine; **PD** Percentage Difference; **SEM** Standard Error of Mean.

Table S58 Ester Linked PC Fatty Acids in Control and HD Cerebellum.

	CON Mean	SEM	N	HD Mean	SEM	N	PD (%)	p	q	Discovery?
**16:0**	568.25	57.39	12	659.62	51.01	13	16.08	0.9357	0.9779	No
**16:1**	2706.37	249.06	12	2490.81	203.20	13	-7.96	0.2465	0.8978	No
**18:0**	7826.81	774.38	12	8373.12	653.47	13	6.98	0.5095	0.9345	No
**18:1**	259.29	23.53	12	341.14	44.14	13	31.57	0.7283	0.9997	No
**18:2**	174.82	19.04	12	178.12	18.68	13	1.89	0.5382	0.9937	No
**20:3**	1504.47	126.50	12	1399.68	68.25	13	-6.96	0.9026	0.9779	No
**20:4**	67.78	8.57	12	87.24	11.95	13	28.72	0.4759	0.9345	No
**20:5**	85.31	8.35	12	92.51	10.50	13	8.44	0.3475	0.8858	No
**22:4**	87.54	8.99	12	92.74	8.91	13	5.95	0.5966	0.9345	No
**22:5**	960.06	91.10	12	792.86	62.76	13	-17.42	0.6846	0.9345	No
**22:6**	107.94	8.76	12	130.08	9.22	13	20.51	0.1465	0.8978	No

Abbreviations: CON Control; **HD** Huntington's disease; **PC** Phosphatidylcholine; **PD** Percentage Difference; **SEM** Standard Error of Mean.

Table S59 Ether Linked PC Fatty Acids in Control and HD Cerebellum.

	CON			HD						
	**Mean**	**SEM**	**N**	**Mean**	**SEM**	**N**	**PD (%)**	**p**	**q**	**Discovery?**
**O-16:0**	107.94	8.76	12	130.08	9.22	13	20.51	0.0868	0.4222	No
**O-16:1**	32.27	3.45	12	48.59	11.94	13	50.55	0.2104	0.8978	No
**O-18:0**	48.73	5.93	12	76.22	8.69	13	56.40	0.0257	0.4222	No
**O-18:1**	180.04	18.75	12	237.26	26.31	13	31.78	0.0909	0.8978	No

Abbreviations: CON Control; **HD** Huntington's disease; **PC** Phosphatidylcholine; **PD** Percentage Difference; **SEM** Standard Error of Mean.

Table S60 Ester PE Species in Control and HD Cerebellum.

	CON Mean	SEM	N	HD Mean	SEM	N	PD (%)	p	q	Discovery?
**PE 16:0_18:1**	266.02	27.80	12	284.60	22.78	13	6.99	0.6104	0.9345	No
**PE 16:1_18:0**	17.21	2.37	12	18.94	1.58	13	10.07	0.5495	0.9345	No
**PE 16:0_18:2**	6.40	0.55	12	9.08	1.72	13	41.83	0.3203	0.8858	No
**PE 16:1_18:1**	42.29	5.70	12	43.69	4.51	13	3.31	0.8492	0.9779	No
**PE 18:0_18:1**	253.54	25.63	12	267.62	29.35	13	5.55	0.9787	>0.9999	No
**PE 18:0_18:2**	21.99	2.66	12	27.37	4.01	13	24.51	0.6114	0.9937	No
**PE 18:1_18:1**	362.52	45.09	12	389.78	42.34	13	7.52	0.9362	>0.9999	No
**PE 16:0_20:3**	8.57	2.55	8	7.19	1.34	12	-16.06	0.6425	0.9345	No
**PE 18:1_18:2**	24.45	3.56	12	32.31	6.31	13	32.12	0.5743	0.9937	No
**PE 16:0_20:4**	2.16	0.41	12	2.71	0.71	13	25.76	>0.9999	>0.9999	No
**PE 18:2_18:2**	109.43	10.06	12	114.99	7.91	13	5.07	0.6686	0.9345	No
**PE 16:0_20:5**	1.47	0.26	8	1.55	0.32	11	5.85	0.8404	>0.9999	No
**PE 16:1_20:4**	9.35	0.83	8	8.32	1.08	11	-11.00	0.4608	0.9345	No
**PE 18:0_20:3**	102.36	13.41	12	108.87	9.51	13	6.36	0.6964	0.9345	No
**PE 16:0_22:4**	49.01	6.22	12	60.07	6.07	13	22.56	0.2159	0.8978	No
**PE 18:0_20:4**	1096.58	113.90	12	982.06	73.21	13	-10.44	0.4082	0.9345	No
**PE 18:1_20:3**	27.70	3.39	12	35.89	5.28	13	29.57	0.4371	0.9937	No
**PE 16:0_22:5**	82.17	8.72	12	98.30	7.62	13	19.63	0.1774	0.8978	No
**PE 18:0_20:5**	5.20	0.69	12	5.11	0.87	13	-1.67	0.5743	0.9937	No
**PE 18:1_20:4**	119.54	15.15	12	128.25	14.19	13	7.29	0.6786	0.9345	No
**PE 18:2_20:3**	1.23	0.22	11	1.63	0.22	12	32.56	0.2604	0.8858	No
**PE 16:0_22:6**	401.13	42.93	12	365.83	27.99	13	-8.80	0.4992	0.9345	No
**PE 16:1_22:5**	11.07	1.47	12	16.16	1.97	13	45.88	0.0398	0.4222	No
**PE 18:1_20:5**	13.72	1.86	12	17.47	2.20	13	27.33	0.2059	0.8978	No
**PE 18:2_20:4**	10.69	0.96	12	13.81	1.26	13	29.19	0.0678	0.4222	No
**PE 16:1_22:6**	15.28	1.87	11	14.02	1.17	10	-8.25	0.5749	0.9345	No
**PE 18:0_22:4**	254.63	27.85	12	286.52	30.00	13	12.52	0.4440	0.9345	No
**PE 18:0_22:5**	152.99	24.01	12	160.13	26.59	13	4.67	0.8517	>0.9999	No
**PE 18:1_22:4**	50.18	7.04	12	65.04	9.58	13	29.61	0.2246	0.8978	No
**PE 18:0_22:6**	1994.73	210.86	12	1731.55	154.11	13	-13.19	0.2945	0.8858	No
**PE 18:1_22:5**	48.35	6.13	12	67.94	5.85	13	40.53	0.0301	0.6945	No

Table S60 Continued… Ester PE Species in Control and HD Cerebellum.

	CON			HD						
	Mean	SEM	N	Mean	SEM	N	PD (%)	p	q	Discovery?
**PE 18:1_22:6**	200.49	22.64	12	199.52	14.06	13	-0.48	0.8100	>0.9999	No
**PE 18:2_22:6**	11.96	1.23	12	13.81	1.11	12	15.45	0.2776	0.9270	No
**PE 20:4_22:6**	21.15	2.09	11	14.50	1.65	13	-31.46	0.0213	0.6945	No
**PE 20:3_22:6**	43.37	6.78	12	37.25	3.88	13	-14.10	0.4439	0.9345	No

Abbreviations: CON Control; **HD** Huntington's disease; **PD** Percentage Difference; **PE** Phosphatidylethanolamine; **SEM** Standard Error of Mean.

Table S61 Ether PE Species in Control and HD Cerebellum.

	CON			HD						
	**Mean**	**SEM**	**N**	**Mean**	**SEM**	**N**	**PD (%)**	**p**	**q**	**Discovery?**
**PE O-18:1_16:0**	110.61	14.35	12	124.59	14.55	13	12.64	0.5009	0.9345	No
**PE O-16:1_18:1**	151.92	22.04	12	173.06	29.51	13	13.92	0.7283	0.9997	No
**PE O-18:2_16:0**	26.44	4.98	12	38.16	8.73	13	44.31	0.3203	0.8858	No
**PE O-18:1_18:1**	155.57	21.41	12	178.16	28.25	13	14.52	0.9787	>0.9999	No
**PE O-18:1_18:2**	223.18	49.58	10	328.59	72.57	12	47.23	0.3136	0.8858	No
**PE O-16:1_20:3**	47.12	6.82	12	80.49	8.43	13	70.82	0.0054	0.4651	No
**PE O-16:1_20:4**	66.52	5.83	12	71.36	7.08	13	7.27	0.9787	>0.9999	No
**PE O-18:0_20:4**	87.19	17.45	11	170.05	28.73	13	95.03	0.0548	0.4222	No
**PE O-18:1_20:3**	244.78	28.42	12	304.94	26.59	13	24.58	0.1360	0.8978	No
**PE O-16:1_22:4**	64.21	10.43	12	96.01	14.80	13	49.53	0.0934	0.8978	No
**PE O-18:1_20:4**	197.37	22.72	12	220.43	16.94	13	11.68	0.4251	0.9345	No
**PE O-18:2_20:3**	15.13	2.82	12	23.11	3.86	13	52.70	0.1683	0.6437	No
**PE O-16:1_22:5**	76.04	9.59	12	89.51	12.28	13	17.72	0.3965	0.9345	No
**PE O-18:1_20:5**	9.12	2.89	12	7.00	1.69	13	-23.31	0.6114	0.9937	No
**PE O-18:2_20:4**	569.78	67.32	12	687.37	71.86	13	20.64	0.2446	0.8978	No
**PE O-16:1_22:6**	153.55	17.33	12	157.29	12.66	13	2.43	0.8635	0.9779	No
**PE O-18:1_22:4**	252.39	31.07	12	342.32	43.90	13	35.63	0.0768	0.4222	No
**PE O-18:0_22:6**	366.66	47.95	12	345.01	45.67	13	-5.91	0.7466	0.9345	No
**PE O-18:1_22:5**	266.35	24.80	12	247.03	28.81	13	-7.25	0.6162	0.9345	No
**PE O-18:2_22:4**	482.24	71.19	12	546.34	71.31	13	13.29	0.5309	0.9345	No
**PE O-18:1_22:6**	570.31	64.81	12	502.26	43.64	13	-11.93	0.3944	0.9345	No
**PE O-18:2_22:5**	9.77	1.48	12	16.78	1.88	13	71.73	0.0077	0.4651	No
**PE O-18:2_22:6**	105.97	14.02	12	123.88	19.63	13	16.90	0.6114	0.9937	No

Abbreviations: CON Control; **HD** Huntington's disease; **PD** Percentage Difference; **PE** Phosphatidylethanolamine; **SEM** Standard Error of Mean.

Table S62 Ester Linked PE Fatty Acids in Control and HD Cerebellum.

	CON			HD						
	**Mean**	**SEM**	**N**	**Mean**	**SEM**	**N**	**PD (%)**	**p**	**q**	**Discovery?**
**16:0**	950.63	100.37	12	991.29	63.16	13	4.28	0.7355	0.9345	No
**16:1**	90.82	11.98	12	96.62	9.07	13	6.38	0.7036	0.9345	No
**18:0**	3899.22	401.29	12	3588.17	284.95	13	-7.98	0.5345	0.9345	No
**18:1**	1716.28	198.32	12	1883.34	148.51	13	9.73	0.5076	0.9345	No
**18:2**	481.46	66.92	12	630.10	65.85	13	30.87	0.0678	0.4222	No
**20:3**	487.31	55.19	12	598.70	34.58	13	22.86	0.1037	0.8978	No
**20:4**	2168.19	232.55	12	2297.58	168.79	13	5.97	0.6573	0.9345	No
**22:4**	1152.66	144.28	12	1396.29	139.87	13	21.14	0.2377	0.8978	No
**22:5**	646.74	65.99	12	695.85	66.19	13	7.59	0.6043	0.9345	No
**22:6**	3881.57	407.60	12	3500.62	254.77	13	-9.81	0.4380	0.9345	No

Abbreviations: CON Control; **HD** Huntington's disease; **PD** Percentage Difference; **PE** Phosphatidylethanolamine; **SEM** Standard Error of Mean.

Table S63 Ether Linked PE Fatty Acids in Control and HD Cerebellum.

	CON			HD						
	**Mean**	**SEM**	**N**	**Mean**	**SEM**	**N**	**PD (%)**	**p**	**q**	**Discovery?**
**O-16:1**	559.36	59.29	12	667.71	50.98	13	19.37	0.1796	0.8978	No
**O-18:0**	446.59	59.94	12	515.06	69.22	13	15.33	0.4622	0.9345	No
**O-18:1**	1992.48	210.78	12	2230.03	202.30	13	11.92	0.4246	0.9345	No
**O-18:2**	1209.34	154.37	12	1435.64	157.34	13	18.71	0.3153	0.9345	No

Abbreviations: CON Control; **HD** Huntington's disease; **PD** Percentage Difference; **PE** Phosphatidylethanolamine; **SEM** Standard Error of Mean.

Table S64 PS Species in Control and HD Cerebellum.

	CON Mean	SEM	N	HD Mean	SEM	N	PD (%)	p	q	Discovery?
**PS 16:0_18:1**	131.24	14.48	12	122.56	7.17	13	-6.61	0.5984	0.9345	No
**PS 18:0_18:1**	1057.94	147.43	12	1401.58	267.59	13	32.48	0.6114	0.9937	No
**PS 18:0_18:2**	14.37	2.37	12	15.49	2.31	13	7.83	0.7373	0.9345	No
**PS 18:1_18:1**	387.11	46.19	12	390.93	53.33	13	0.99	0.9573	0.9917	No
**PS 16:0_20:3**	11.50	1.03	7	12.29	1.10	8	6.80	0.6126	0.9937	No
**PS 18:0_20:3**	115.83	13.20	12	116.60	11.26	13	0.66	0.9653	0.9917	No
**PS 18:0_20:4**	100.85	11.91	12	118.20	12.29	13	17.21	0.3213	0.9345	No
**PS 18:1_20:4**	21.03	4.09	8	22.52	2.21	13	7.10	0.7542	0.9345	No
**PS 16:0_22:6**	17.24	1.61	8	17.70	1.61	8	2.65	0.8440	0.9779	No
**PS 18:0_22:4**	196.93	25.01	12	219.85	26.68	13	11.64	0.5370	0.9345	No
**PS 18:0_22:5**	136.01	21.75	12	159.06	28.72	13	16.95	0.7283	0.9997	No
**PS 18:0_22:6**	1370.15	146.44	12	1330.88	101.93	13	-2.87	0.9362	>0.9999	No

Abbreviations: CON Control; **HD** Huntington's disease; **PD** Percentage Difference; **PS** Phosphatidylserine; **SEM** Standard Error of Mean.

Table S65 PS Fatty Acids in Control and HD Cerebellum.

	CON Mean	SEM	N	HD Mean	SEM	N	PD (%)	p	q	Discovery?
**16:0**	149.45	17.46	12	141.01	9.25	13	-5.64	0.6750	0.9345	No
**18:0**	2992.07	342.85	12	3361.66	367.82	13	12.35	0.4698	0.9345	No
**18:1**	1977.42	249.78	12	2328.53	373.97	13	17.76	0.7283	0.9997	No
**18:2**	14.37	2.37	12	15.49	2.31	13	7.83	0.7373	0.9345	No
**20:3**	122.54	14.18	12	124.16	12.32	13	1.32	0.9323	0.9779	No
**20:4**	114.86	15.20	12	140.72	14.13	13	22.51	0.2255	0.8978	No
**22:4**	196.93	25.01	12	219.85	26.68	13	11.64	0.5370	0.9345	No
**22:5**	136.01	21.75	12	159.06	28.72	13	16.95	0.7283	0.9997	No
**22:6**	1381.64	148.63	12	1341.78	103.16	13	-2.89	0.9787	>0.9999	No

Abbreviations: CON Control; **HD** Huntington's disease; **PD** Percentage Difference; **PS** Phosphatidylserine; **SEM** Standard Error of Mean.

Table S66 Total Ester Linked Phospholipid Derived Fatty Acids in Control and HD Cerebellum.

	CON			HD						
	**Mean**	**SEM**	**N**	**Mean**	**SEM**	**N**	**PD (%)**	**p**	**q**	**Discovery?**
**16:0**	13761.69	1307.70	12	13914.04	902.67	13	-15.18	0.9246	0.9779	No
**16:1**	659.07	68.85	12	756.23	48.27	13	-23.41	0.2614	0.9242	No
**18:0**	9597.66	977.93	12	9440.64	706.52	13	-31.65	0.8977	0.9779	No
**18:1**	11520.52	1207.81	12	12584.99	1070.34	13	-39.89	0.5162	0.9345	No
**18:2**	755.12	82.87	12	986.74	83.43	13	-48.13	0.0611	0.8978	No
**20:3**	784.67	85.11	12	900.97	44.34	13	-56.37	0.2425	0.8978	No
**20:4**	3787.53	367.77	12	3837.98	219.91	13	-64.60	0.9076	0.9779	No
**20:5**	67.78	8.57	12	87.24	11.95	13	-63.60	0.1995	0.8978	No
**22:4**	1434.89	175.83	12	1708.65	170.94	13	-72.84	0.2759	0.9270	No
**22:5**	870.28	92.78	12	947.65	88.52	13	-81.08	0.5522	0.9345	No
**22:6**	6223.27	639.54	12	5635.25	384.58	13	-89.32	0.4409	0.9345	No
**Total Saturated**	23359.35	2275.16	12	23354.68	1583.73	13	-0.02	0.9987	>0.9999	No
**Total Monounsaturated**	12179.59	1275.12	12	13341.22	1099.91	13	9.54	0.4975	0.9345	No
**Total Polyunsaturated**	13923.54	1393.06	12	14104.49	878.11	13	1.30	0.9137	0.9779	No
**Total Phospholipid**	49462.48	4929.87	12	50800.39	3394.51	13	2.70	0.8254	0.9779	No

Abbreviations: CON Control; **HD** Huntington's disease; **PD** Percentage Difference; **SEM** Standard Error of Mean.

Table S67 Phospholipid Class Totals in Control and HD Cerebellum.

	CON			HD						
	**Mean**	**SEM**	**N**	**Mean**	**SEM**	**N**	**PD (%)**	**p**	**q**	**Discovery?**
**Total Ester PC**	13285.99	1251.90	12	13420.99	902.97	13	1.02	0.9312	0.9779	No
**Total Ether PC**	368.99	31.65	12	492.14	44.46	13	33.38	0.0347	0.6945	No
**Total Ester PE**	5829.33	612.97	12	5625.40	413.87	13	-3.50	0.7856	0.9538	No
**Total Ether PE**	4207.76	463.00	12	4848.44	426.84	13	15.23	0.3197	0.9345	No
**Total PS**	3542.65	406.07	12	3916.13	427.30	13	10.54	0.5326	0.9345	No

Abbreviations: CON Control; **HD** Huntington's disease; **PC** Phosphatidylcholine; **PD** Percentage Difference; **PE** Phosphatidylethanolamine;
PS Phosphatidylserine;
SEM Standard Error of Mean.

Table S68 LPC and LPE Species in Control and HD Cerebellum.

Cerebellum	CON Mean	SEM	N	HD Mean	SEM	N	PD (%)	p	q	Discovery?
**LPC 16:0**	9.68	3.25	9	8.27	2.73	7	-14.62	>0.9999	>0.9999	No
**LPC 16:1**	1.08	0.18	9	2.18	1.36	6	101.87	0.95	>0.9999	No
**LPC 18:0**	3.54	0.96	6	4.15	2.07	3	17.42	0.90	>0.9999	No
**LPC 18:1**	9.08	1.99	7	10.46	5.19	5	15.19	0.60	>0.9999	No
**LPC 18:2**	0.56	0.08	8	1.14	0.74	8	103.64	0.72	>0.9999	No
**LPC 20:4**	3.99	1.10	9	0.79	0.36	7	-80.26	0.01	0.10	No
**LPC 22:6**	2.85	0.59	9	3.06	0.54	3	7.32	0.73	>0.9999	No
**Total LPC**	22.52	6.75	11	15.95	6.95	10	-29.16	0.28	>0.0001	No
**LPE 16:0**	11.73	2.45	5	14.36	5.27	7	22.38	>0.9999	>0.9999	No
**LPE 16:1**	2.87	0.78	6	7.45	1.54	6	159.66	0.03	0.17	No
**LPE 16:2**	5.73	1.23	7	7.17	1.88	7	25.30	0.62	>0.9999	No
**LPE 18:0**	17.76	5.57	9	16.17	3.89	9	-8.97	0.82	0.89	No
**LPE 22:5**	6.36	1.88	6	12.46	4.96	6	95.88	0.59	>0.9999	No
**LPE 22:6**	78.74	23.83	7	82.73	11.52	7	5.06	0.53	>0.0001	No
**Total LPE**	78.65	22.48	11	82.90	17.78	12	5.40	0.88	0.89	No

Abbreviations: CON Control; **HD** Huntington's disease; **LPC** Lysophosphatidylcholine; **LPE** Lysophophatidylethanolamine; **PD** Percentage Difference; **SEM** Standard Error of Mean
